# Changes in the management of hypertension, diabetes mellitus, and hypercholesterolemia in Korean adults before and during the COVID-19 pandemic: data from the 2010-2020 Korea National Health and Nutrition Examination Survey

**DOI:** 10.4178/epih.e2023014

**Published:** 2023-02-01

**Authors:** Yoonjung Kim, Suyeon Park, Kyungwon Oh, Hongseok Choi, Eun Kyeong Jeong

**Affiliations:** 1 Division of Health and Nutrition Survey and Analysis, Bureau of Chronic Disease Prevention and Control, Korea Disease Control and Prevention Agency, Cheongju, Korea; 2 Bureau of Chronic Disease Prevention and Control, Korea Disease Control and Prevention Agency, Cheongju, Korea; 3 Public Health Care Headquarters, Seoul National University Bundang Hospital, Seongnam, Korea

**Keywords:** COVID-19 pandemic, Hypertension, Diabetes mellitus, Hypercholesterolemia

## Abstract

**OBJECTIVES:**

This study aimed to analyze the changes in chronic disease management indicators, including hypertension, diabetes mellitus, and hypercholesteremia, from 2010-2020 and before (2019) and during (2020) the coronavirus disease 2019 (COVID-19) pandemic.

**METHODS:**

This study included 58,504 individuals aged ≥30 years who participated in the Korea National Health and Nutrition Examination Survey 2010-2020. Trends in the prevalence, awareness, treatment, and control of chronic diseases and the difference in those between before and during the COVID-19 pandemic were analyzed using the SAS program PROC SURVEYREG.

**RESULTS:**

From 2010-2020, the awareness, treatment, and control in adults aged ≥30 years for hypertension and hypercholesterolemia continuously improved, whereas no significant change in the management indicators of diabetes mellitus was observed. The prevalence of hypertension, diabetes mellitus, and hypercholesterolemia in men increased from before to during the COVID-19 pandemic. However, there was no significant change in the management indicators of hypertension and diabetes mellitus in men and women, and the awareness, treatment, and control rates for hypercholesterolemia increased by 5.5%p, 6.9%p, and 4.1%p respectively.

**CONCLUSIONS:**

In 2020, the first year of the COVID-19 pandemic, the prevalence of hypertension, diabetes mellitus, and hypercholesterolemia increased, but the management indicators of the chronic diseases did not significantly deteriorate. Considering the ongoing COVID-19 pandemic, it is necessary to monitor changes in chronic disease management indicators and to develop efficient and accessible chronic disease prevention and management programs.

## INTRODUCTION

In 2020, the mortality rates of heart disease, cerebrovascular disease, diabetes mellitus, and hypertensive disease in Korea were 10.6% (second-leading cause of death), 7.2% (fourth-leading cause of death), 2.8% (sixth-leading cause of death), and 2.0% (the ninthleading cause of death), respectively. Cardiocerebrovascular disease is not only a major cause of death, it also has a high disease burden. Thus, the prevention and management of cardiocerebrovascular diseases is very important [1]. For those aged 30 years or older, the prevalence of hypertension, diabetes mellitus, and hypercholesterolemia, which are major underlying diseases of cerebrocardiovascular disease, are 34.2%, 16.7%, and 27.5%, respectively, which are very high [2]. Thus, it is necessary to properly manage these diseases through an early diagnosis by periodic health examinations, the continual use of medication, and the improvement of health behaviors, such as physical activity and eating habits.

To reduce the prevalence of hypertension, diabetes mellitus, and dyslipidemia, which are the underlying diseases of four chronic diseases with high disease burdens (cardiocerebrovascular disease, diabetes mellitus, chronic respiratory disease, and cancer) and to improve the management of chronic diseases, Korea established the Health Plan 2030, is proceeding with health policies and interventions, and is monitoring the progress in achieving improved outcomes through the Korea National Health and Nutrition Examination Survey (KNHANES) [3].

As chronic diseases have been reported as risk factors for severe illness and complications associated with coronavirus disease 2019 (COVID-19), the importance of chronic disease management has been further highlighted. People with chronic diseases were vulnerable to COVID-19, and those with uncontrolled chronic diseases who contracted COVID-19 were more likely to develop severe symptoms. In Korea, chronic diseases such as hypertension and diabetes mellitus were the most common underlying diseases in persons aged 50 years or older who were hospitalized with COVID-19 [4]. In the United States, 94.9% of patients who were hospitalized with COVID-19 had at least one underlying disease, the most common of which were hypertension and lipid metabolism disorders. In addition, those with one chronic disease were 1.32 times more likely to be admitted to an intensive care unit and 1.53 times more likely to die compared with those without a chronic disease [5].

In Korea, there were 24,709,789 patients with confirmed COVID-19 (47.9% of the population) as of August 2022. Many of these patients were hospitalized and treated for severe illness, and 28,318 of these patients died as of September 28, 2022. It is assumed that the COVID-19 pandemic may have a negative impact on the prevention and management of chronic diseases due to the COVID-19-related restrictions on the use of medical services, as well as direct health damage, such as an increase in the prevalence of chronic diseases, such as obesity and hypertension, in adults [6]. The diagnosis of chronic diseases may be delayed due to a decline in health examinations, and regular treatments and medications in patients with chronic diseases may be restricted due to the selfquarantine of patients with confirmed COVID-19 and their contacts and their avoidance of hospital visits, which is likely to reduce the treatment of chronic diseases. In addition, there is a possibility that the chronic disease management indicators may be deteriorating due to various factors, such as a reduction in chronic disease prevention and management services provided by the private and public sectors, a deterioration of health behaviors caused by social distancing, such as a decrease in physical activity, an increased consumption of delivery food or instant food, and an increase in stress levels [6-10]. Therefore, it is important to investigate the effects of the COVID-19 pandemic on chronic disease management indicators and to develop countermeasures to strengthen the management of chronic diseases.

This study analyzed changes in the indicators of hypertension, diabetes mellitus, and hypercholesterolemia management between before (2019) and during (2020) the COVID-19 pandemic to provide evidence for the management of chronic diseases after the COVID-19 pandemic. In addition, this study analyzed the trends in chronic disease management indicators over the past 11 years (2010-2020) to identify differences between the changes due to the COVID-19 pandemic and long-term changes.

## MATERIALS AND METHODS

### Participants

The KNHANES is a national health survey conducted each year by the Korea Disease Control and Prevention Agency (KDCA) that has been assessing the health and nutritional status of Koreans since 1998. To represent the entire Korean population, approximately 200 primary sampling units (PSUs) with 20-25 households per PSU in each year survey were sampled using a stratified two-stage cluster sampling method. The participants of the KNHANES each year includes about 10,000 individuals or all household members aged 1 year or older in the sampled households. Among those who participated the health examination and health interview in the KNHANES 2010-2020, those aged 30 years or older were analyzed in this study. The number and general characteristics of the participants are shown in Table 1.

## Methods

The KNHANES health examinations (blood pressure measurement, blood sampling, etc.) and health interviews were conducted by trained interviewers at the mobile examination center (MEC). Blood pressure was measured three times in the right arm in a sitting position after resting for 5 minutes using a mercury sphygmomanometer in 2010-2019 (Baumanometer; Baum, Copiague, NY, USA) or Accoson Greenlight 300^TM^ (Greenlight; Accoson, Essex, UK) in 2020, and the mean value of the second and third blood pressure measurement values was used. To calculate the prevalence of diabetes mellitus and hypercholesterolemia, blood samples were collected from each participant, and the blood samples collected from only those who had fasted for at least 8 hours were used in the analysis. After all specimens were processed and refrigerated in MEC, they were sent to clinical labs for analysis on the same day. Data on glycated hemoglobin (HbA1c) levels have been collected since 2011, when HbA1c in all persons aged 10 years or older began to be analyzed. The quality control of the blood pressure measurements was conducted in collaboration with the Korean Society of Hypertension, and the quality control of clinical laboratory tests was conducted in collaboration with the Korean Society for Laboratory Medicine. Each participant’s experiences of receiving a diagnosis in his/her lifetime and current taking medication, which are required to calculate the prevalence and management indicators of chronic disease, were investigated through interviews.

Hypertension was defined as a systolic blood pressure of ≥ 140 mmHg, a diastolic blood pressure of ≥ 90 mmHg or the use of antihypertensive drugs. Diabetes mellitus was defined as a fasting blood glucose of ≥ 126 mg/dL or HbA1c of ≥ 6.5%, or a diagnosis of diabetes mellitus by a doctor, or the use of hypoglycemic drugs or insulin injections according to the criteria by the Korean Diabetes Association. Hypercholesterolemia was defined as a total blood cholesterol level of ≥ 240 mg/dL or the use of cholesterol-lowering drugs. The awareness rates of hypertension, diabetes mellitus, and hypercholesterolemia were calculated as the percentages of those who reported being diagnosed with the disease by a doctor for each respective disease. The treatment rates of hypertension, diabetes mellitus, and hypercholesterolemia were calculated as the percentages of those who were currently taking medications for those with each respective disease. The control rate was defined as the percentage of those who reached the treatment goals (blood pressure <140/90 mmHg, HbA1c <6.5%, or blood cholesterol <200 mg/dL) of those with hypertension, diabetes mellitus, or hypercholesterolemia who were undergoing treatment.

### Statistical analysis

The data analysis was performed using SAS, version 9.4 (SAS Institute Inc., Cary, NC, USA). To best represent the characteristics of Koreans as the target population of the KNHANES, all results were calculated with weights using analysis methods for complex sampling data. The differences in the distributions of gender, age, and household income level of the participants were analyzed using the Rao–Scott chi-square test. The prevalence and management indicators of chronic disease were presented as crude values and were calculated using PROC SURVEYMEANS. To compare the trends in the chronic disease management indicators by year, age-standardized rates should be presented. However, because age group-specific data for some chronic disease management indicators are not sufficient, crude values are presented. The statistical significance for trend analysis from 2010 to 2020 and between 2010 and 2019, and the differences between 2019 and 2020 was performed using PROC SURVEYREG after adjusting for age and household income level. Because it was difficult to secure an appropriate sample size with only the data of the relevant year, it was analyzed by classifying the survey periods (2010-2012, 2013-2015, 2016-2018, and 2019-2020) and the age group into those aged 30-64 years and those aged 65 years or older.

### Ethics statement

This study was approved by the Institutional Review Board (IRB) of the KDCA in accordance with the annual survey plans (2010-2014 and 2018-2020), and it received an exemption from the IRB review for three years (2015-2017) in accordance with the Bioethics and Safety Act, Article 2 (2) and the Enforcement Regulations, Article 2 (2-1).

## RESULTS

The general characteristics of the participants are shown in Table 1. There was a total of 58,504 participants aged 30 years or older. The number of men and women aged 30-64 years decreased, whereas the number of those aged 65 or older increased. The number of women with a middle household income or below decreased.

As of 2020, the prevalence of hypertension (crude, age ≥30 years) in men was 38.9%, and that in women was 29.7%. The prevalence of hypertension increased in men and decreased in women over the past 11 years (2010-2020). The awareness, treatment, and control of hypertension were 71.3%, 66.8%, and 72.2%, respectively, as of 2019-2020. Over the past 11 years (2010-2020), the awareness and treatment of hypertension in men tended to increase in those aged 30-64 years, and the awareness of hypertension in women decreased in only those aged 30-64 years. The control of hypertension among treated persons increased in men aged 30 years or older and women aged 30-64 years. When comparing between before (2019) and during (2020) the COVID-19 pandemic, the prevalence of hypertension increased significantly in only men aged 30-64 years (28.9% in 2019 vs. 34.4% in 2020), and there was no statistically significant change in the awareness and treatment of hypertension and in the control of hypertension in the treated persons (Table 2).

As of 2020, the prevalence of diabetes mellitus (crude, age ≥ 30 years) in men was 19.2%, and that of women was 14.3%. Over the past 10 years (2011-2020), the prevalence of diabetes mellitus in men increased, whereas no change was observed in women. The awareness, treatment, and control rates of diabetes mellitus were 65.8%, 61.4%, and 25.2%, respectively, as of 2019-2020. Over the past 10 years (2011-2020), the awareness and treatment of diabetes mellitus showed no significant change in men and markedly increased only in women aged 65 years or older. The control of diabetes mellitus in treated persons has been around 25%, and it has not significantly changed over the past 10 years. When comparing before (2019) and after (2020) the COVID-19 pandemic, the prevalence of diabetes mellitus increased in only men aged 30-64 years, and the awareness, treatment, and control rates showed no change in men and women (Table 3).

As of 2020, the prevalence of hypercholesterolemia (crude, age ≥ 30 years) in men was 26.4%, and that in women was 28.6%. The awareness and treatment of hypercholesterolemia and the control of hypercholesterolemia in treated persons were 64.6%, 56.7%, and 85.1%, respectively, as of 2019-2020. Over the last 11 years (2010-2020), the prevalence of hypercholesterolemia increased by 12%p in both men and women, and the awareness and treatment of hypercholesterolemia increased by about 20%p, respectively. The control rate of hypercholesterolemia in treated persons increased by about 6%p. When comparing before (2019) and after (2020) the COVID-19 pandemic, the prevalence of hypercholesterolemia increased in only men aged 30-64 years. However, the awareness and treatment of hypercholesterolemia increased in only men aged 65 years and older, and the control rate among treated persons increased in only women aged 30-64 years (Table 4).

For the health behaviors in those with hypertension, diabetes mellitus, and hypercholesterolemia over the past 11 years (2010-2020), the current smoking rate and excessive sodium intake decreased. Meanwhile, the prevalence of obesity (men), high-risk alcohol drinking rate (men), aerobic physical activity rate (both men and women), and perceived stress (men) tended to worsen and were more distinct in men than in women. Comparing before (2019) and after (2020) the COVID-19 pandemic, the prevalence of obesity increased in men with hypertension aged 30-64 years, and the high-risk alcohol drinking rate increased in men with hypercholesterolemia aged 30-64 years (Supplementary Materials 1-3).

## DISCUSSION

The results of the analysis of changes in the chronic disease management indicators in adults using the data from the KNHANES 2010-2020 revealed that the awareness, treatment, and control of hypertension and hypercholesterolemia have continued to improve over the last 11 years (2010-2020), whereas there has been no significant change in the diabetes mellitus management indicators over the same period. These results are not different from the trends in the chronic disease management indicators between 2005 and 2018, as demonstrated in a previous study [11]. There was no significant change in the hypertension and diabetes mellitus management indicators from before to during the COVID-19 pandemic, whereas there was an improvement in the hypercholesterolemia management indicators.

To increase the awareness of chronic diseases, an early diagnosis of these diseases is important, and health examinations for hypertension, diabetes mellitus, dyslipidemia, etc., in Korea are conducted every 1-2 years under the national health screening. In 2020, the first year of the COVID-19 outbreak, the health examination rate was 67.8%, a 6.3%p decrease from the 74.1% reported in 2019 [12]. In this study, there was no change in the awareness of hypertension and diabetes mellitus observed in 2020, during the COVID-19 pandemic. A decrease in the general health examination rate in 2020 did not result in a decrease in the awareness of major chronic diseases. However, it is necessary to monitor health examination and the awareness of chronic diseases in settings where the COVID-19 pandemic persists or a long time.

Regular medical treatment and continual medication use are important for the proper management of chronic diseases. According to the national health insurance statistics in Korea, the average number of hospitalizations and hospital visits per person per month was 1.74 days in 2019, but the number of days of healthcare utilization decreased after the COVID-19 pandemic (1.56 days in 2020, and 1.55 days in 2021). In addition, concerning the number of those receiving healthcare service in 2020 compared with that in 2019, that for hypertension (n = 6.73 million) and diabetes mellitus (n = 3.34 million) increased by 3.0%, and 3.6%, respectively, which were lower than the average annual increase rates of 3.3% and 5.4%, respectively, indicating that it is presumed that the healthcare utilization for hypertension and diabetes mellitus may be restricted [13]. In particular, the number of patients who received healthcare service for hypertension and diabetes mellitus from March 2020 to July 2020 at the beginning of the COVID-19 pandemic increased compared with the same period in 2019, but the percent change in 2020 compared with 2016-2019 decreased by 0.2% and 2.4%, respectively [14]. In this study, no change in the treatment for hypertension and diabetes mellitus in 2019 and 2020 was observed, indicating that, despite restrictions on healthcare utilization due to the COVID-19 pandemic, their impact on the treatment was minimal. This may be due to the fact that medications by proxy or telephone consultations were temporarily allowed during the COVID-19 pandemic (effective starting February 24, 2020), and the prescriptions of drugs thus continued. Similar to the results of this study, studies have reported that the appropriate medication administration rate for hypertension in 2020 (rate of drug prescription for ≥ 290 days: 60.6%) was not different from that in 2019 (59.9%) [15] and 91.9% of patients with hypertension and 94.6% of patients with diabetes mellitus did not attended or postponed an outpatient visit for hypertension and diabetes mellitus during the past year [16]. In the United States, telemedicine was strengthened during the COVID-19 pandemic, but 40.9% of those aged 18 years or older (regular medical checkup rate: 31.5%; emergency treatment rate: 12.0%) delayed or avoided medical care due to COVID-19 [17]. From February 2020 to March 2020, at the beginning of the COVID-19 pandemic, the rates of testing for diabetes mellitus and dyslipidemia fell by 81-90%, and the rate of new medication therapy decreased by 52-60% [18].

To prevent complications from chronic diseases, such as hypertension and diabetes mellitus, it is necessary to maintain blood pressure, glucose, and cholesterol levels within normal range through continual medication use, the maintenance of ideal body weight, increasing physical activity, and nutrition management. The control of diabetes mellitus in those undergoing diabetes treatment was lower and did not significantly change over the past 10 years compared with the control of hypertension and hypercholesterolemia, which was evident in men. Because diabetes mellitus is caused by complex factors, it is important to improve its risk factors, such as health behaviors and obesity, along with providing continuous treatment [11,19]. This study demonstrated that, with regard to the changes in risk factors in patients with diabetes mellitus in 2011-2020, obesity, high-risk alcohol drinking, physical activity, and stress levels except for smoking worsened (Supplementary Material 2). In particular, the prevalence of obesity among men with diabetes mellitus increased in those of all ages, and the prevalence of obesity in those aged 30-64 years increased by 16.3%p, from 47.0% in 2011-2012 to 63.3% in 2019-2020. There is a possibility that the control rate of diabetes mellitus may be low due to difficulty in improving health behaviors as shown in the trends in risk factors among those with diabetes mellitus, lack of awareness of drug efficacy, fear of hypoglycemia and drug side effects, and low drug adherence due to cost burden [20,21]. In the United States (1999-2018) [22] and China (2013-2018) [23], the prevalence of diabetes mellitus also increased, but the control of diabetes mellitus did not change, which are findings that are similar to the results of this study. In this study, there was no change in the control of diabetes mellitus between before and during the COVID-19 pandemic, and there was also no significant difference in the health behaviors in patients with diabetes mellitus between 2019 and 2020.

The awareness, treatment, and control of hypercholesterolemia all continuously improved since 2010, which is a trend that is different from those of hypertension and diabetes mellitus. The changes in health behavior in patients with hypercholesterolemia tended to be similar to those with hypertension and those with diabetes mellitus (Supplementary Materials 1-3), and its control rate was likely to be high due to the high efficacy of antidyslipidemia drugs and medication adherence. In the United States, the reasons why the control rate of hypercholesterolemia was high (2017-2018, 79.5%) despite the prevalence of hypercholesterolemia (1988-2018) increasing [24], and other risk factors, such as obesity and blood glucose levels were not improving, are statin therapy, increased medication adherence, and, in particular, the impact of the revised guidelines issued in 2013 by the related society that emphasized the administration of high-dose statins to high-risk groups [25].

Comparing before (2019) and during (2020) the COVID-19 pandemic, the prevalence of hypertension, diabetes mellitus, and hypercholesterolemia increased in men aged 30-64 years, but there was no change in the hypertension and diabetes mellitus management indicators in those requiring management. Previous studies have reported that, after the COVID-19 pandemic, the prevalence of obesity increased and physical activity decreased in those in their thirties and that high-risk alcohol drinking rate increased in those in their forties [6,8,10]. This study also demonstrated that, for men aged 30-64 years regardless of having chronic diseases, obesity and alcohol drinking indicators deteriorated, and intensive health care for this age group is thus required.

Due to the COVID-19 pandemic, other countries, such as the United States and the United Kingdom, suspended or reduced the size of nationwide surveys. Meanwhile, the KNHANES was conducted during the COVID-19 pandemic using the same survey methods (measurements, health examination, etc.) and a similar sample size (survey completion rate: 94%) as those used in previous years, and it has advantages in that it can analyze the health status of the population before and during the COVID-19 pandemic. In addition, to maintain the accuracy of the KNHANES data, the KNHANES is conducted by trained interviewers and medical staff who completed training and passed evaluation for survey guideline, and routine quality control in the KNHANES is conducted in collaboration with related academic societies or professional organizations. However, as the KNHANES is a cross-sectional survey, the data on the chronic disease management indicators and related risk factors from the KNHANES were collected from different persons with chronic diseases each year. Considering this, it is necessary to interpret these trend in chronic disease management indicators, and there is a limitation that it cannot confirm whether the COVID-19 pandemic had a direct impact on changes in health behaviors and the prevalence and management indicators of chronic disease.

## CONCLUSION

Despite the fact that the prevalence of obesity, diabetes mellitus, and hypercholesterolemia in adults increased in 2020, the first year of the COVID-19 pandemic, the chronic disease management indicators did not significantly deteriorate. However, as the COVID-19 pandemic persists for a long time, it is predicted that the worsening of health risk behaviors and increasing prevalence of chronic diseases, such as obesity and hypertension, may affect chronic disease management indicators. Therefore, it is necessary to develop and apply effective chronic disease prevention and management programs, such as education programs based on the nonface-to-face method using online and mobile platforms and the face-to-face method that are easily accessible to the target populations, registry management programs for patients with chronic diseases, and additional public relations activities.

## Figures and Tables

**Table 1.** Characteristics of the study participants, which included Korean men and women aged 30 years or older in the 2010-2020 Korea National Health and Nutrition Examination Survey

Characteristics			2010-2012	2013-2015	2016-2018	2019-2020	2019	2020	Rac-Scott chi-square test p-value	
									2010-2020	2019-2020
Total (≥30 yr)										
	Age (yr)								<0.001	0.574
		Total (≥30)	16,431 (100)	15,042 (100)	16,367 (100)	10,664 (100)	5,543 (100)	5,121 (100)		
		30-64	11,874 (82.1)	10,702 (80.8)	11,563 (79.7)	7,293 (77.9)	3,853 (78.4)	3,440 (77.4)		
		≥65	4,557 (17.9)	4,340 (19.2)	4,804 (20.3)	3,371 (22.1)	1,690 (21.6)	1,681 (22.6)		
	Household income^1^								0.021	0.981
		Low	3,233 (22.4)	2,994 (20.3)	3,240 (20.0)	2,121 (19.5)	1,105 (19.7)	1,016 (19.4)		
		Low-middle	3,263 (21.0)	2,965 (19.9)	3,285 (20.2)	2,124 (19.7)	1,103 (20.1)	1,021 (19.3)		
		Middle	3,239 (19.8)	2,996 (20.0)	3,262 (20.0)	2,125 (20.1)	1,103 (20.0)	1,022 (20.1)		
		Middle-high	3,205 (18.7)	3,023 (20.2)	3,263 (20.1)	2,122 (20.5)	1,101 (20.2)	1,021 (20.7)		
		High	3,254 (18.1)	2,957 (19.6)	3,255 (19.6)	2,121 (20.2)	1,103 (20.0)	1,018 (20.5)		
Men									<0.001	0.568
	Age (yr)									
		Total (≥30)	7,033 (48.9)	6,410 (48.8)	7,121 (49.1)	4,655 (49.1)	2,404 (49.1)	2,251 (49.2)		
		30-64	5,079 (85.0)	4,551 (83.6)	5,047 (82.4)	3,208 (80.6)	1,681 (81.1)	1,527 (80.2)		
		≥65	1,954 (15.0)	1,859 (16.4)	2,074 (17.6)	1,447 (19.4)	723 (18.9)	724 (19.8)		
	Household income^1^								0.450	0.982
		Low	1,381 (22.1)	1,276 (20.4)	1,409 (19.8)	926 (19.7)	483 (19.9)	443 (19.4)		
		Low-middle	1,382 (20.6)	1,275 (20.1)	1,419 (20.2)	928 (19.7)	478 (20.0)	450 (19.5)		
		Middle	1,403 (20.2)	1,274 (20.1)	1,420 (20.3)	923 (20.0)	474 (20.2)	449 (19.8)		
		Middle-high	1,355 (18.6)	1,287 (20.1)	1,421 (20.0)	927 (20.5)	477 (20.2)	450 (20.7)		
		High	1,414 (18.6)	1,254 (19.4)	1,421 (19.7)	934 (20.2)	482 (19.7)	452 (20.6)		
Women										
	Age (yr)								<0.001	0.629
		Total (≥30)	9,398 (51.1)	8,632 (51.2)	9,246 (50.9)	6,009 (50.9)	3,139 (50.9)	2,870 (50.8)		
		30-64	6,795 (79.4)	6,151 (78.1)	6,516 (77.1)	4,085 (75.3)	2,172 (75.8)	1,913 (74.8)		
		≥65	2,603 (20.6)	2,481 (21.9)	2,730 (22.9)	1,924 (24.7)	967 (24.2)	957 (25.2)		
	Household income^1^								0.004	0.974
		Low	1,852 (22.7)	1,718 (20.2)	1,831 (20.3)	1,195 (19.4)	622 (19.4)	573 (19.4)		
		Low-middle	1,881 (21.4)	1,690 (19.6)	1,866 (20.2)	1,196 (19.7)	625 (20.2)	571 (19.2)		
		Middle	1,836 (19.5)	1,722 (20.0)	1,842 (19.8)	1,202 (20.1)	629 (19.9)	573 (20.3)		
		Middle-high	1,850 (18.8)	1,736 (20.3)	1,842 (20.1)	1,195 (20.5)	624 (20.3)	571 (20.7)		
		High	1,840 (17.6)	1,703 (19.9)	1,834 (19.6)	1,187 (20.3)	621 (20.2)	566 (20.4)		

Values are presented as the crude number (weighted %).

1 Calculated as the monthly household income divided by square root of the number of persons in the household, categorized into quantiles according to age and gender.

**Table 2.** Trends in prevalence and management of hypertension among Korean men and women aged 30 years or older in the 2010-2020 Korea National Health and Nutrition Examination Survey

Indicators	Variables		2010-2012	2013-2015	2016-2018	2019-2020	2019	2020	Trend		Difference 2019 to 2020
									2010-2020 (β estimate)	2010-2019 (β estimate)	
Prevalence	Total (yr)										
		Total (≥30)	30.4 (0.6)	30.4 (0.6)	32.7 (0.5)	33.6 (0.7)	32.9 (1.0)	34.2 (1.0)	-0.016	-0.049	1.3
		30-64	23.4 (0.5)	23.1 (0.6)	24.9 (0.5)	25.2 (0.6)	24.2 (0.8)	26.3 (0.9)	-0.003	-0.069	2.1
		≥65	63.3 (0.9)	61.4 (1.0)	63.2 (0.9)	62.9 (1.0)	64.4 (1.4)	61.4 (1.5)	-0.016	0.096	-3.1
	Men (yr)										
		Total (≥30)	32.4 (0.7)	33.7 (0.8)	36.4 (0.7)	36.8 (0.9)	34.7 (1.3)	38.9 (1.2)	0.242^*^	0.148	4.2^*^
		30-64	28.3 (0.8)	29.2 (0.8)	31.4 (0.7)	31.6 (1.0)	28.9 (1.3)	34.4 (1.4)	0.212	0.062	5.5^*^
		≥65	56.1 (1.4)	56.5 (1.5)	59.4 (1.2)	58.1 (1.6)	59.4 (2.2)	56.9 (2.2)	0.384	0.578^*^	-2.5
	Women (yr)										
		Total (≥30)	28.4 (0.7)	27.4 (0.6)	29.1 (0.7)	30.5 (0.8)	31.2 (1.1)	29.7 (1.2)	-0.281^*^	-0.253^*^	-1.5
		30-64	18.4 (0.6)	17.3 (0.6)	18.2 (0.6)	18.7 (0.7)	19.3 (0.9)	18.0 (1.0)	-0.253^*^	-0.231^*^	-1.4
		≥65	68.4 (1.3)	64.9 (1.3)	65.9 (1.2)	66.5 (1.3)	68.2 (1.8)	64.8 (1.7)	-0.276	-0.223	-3.4
Awareness	Total (yr)										
		Total (≥30)	65.9 (1.0)	67.3 (0.9)	69.1 (0.8)	71.3 (0.9)	71.4 (1.3)	71.1 (1.3)	0.257^*^	0.200	-0.3
		30-64	55.6 (1.3)	56.1 (1.3)	58.3 (1.1)	61.6 (1.3)	61.9 (1.9)	61.4 (1.7)	0.372^*^	0.279	-0.5
		≥65	83.9 (1.0)	85.2 (0.8)	85.8 (0.8)	84.9 (0.9)	84.3 (1.1)	85.5 (1.4)	0.140	0.152	1.2
	Men (yr)										
		Total (≥30)	56.9 (1.4)	60.1 (1.4)	64.0 (1.2)	67.1 (1.3)	67.3 (1.9)	67.0 (1.9)	0.759^***^	0.703^*^	-0.2
		30-64	48.4 (1.7)	50.6 (1.7)	55.4 (1.5)	59.3 (1.7)	58.9 (2.6)	59.6 (2.3)	0.915^*^	0.825^*^	0.7
		≥65	81.5 (1.4)	84.8 (1.2)	85.2 (1.1)	84.8 (1.4)	84.6 (1.9)	85.0 (2.2)	0.388	0.461	0.4
	Women (yr)										
		Total (≥30)	75.7 (1.1)	75.4 (1.0)	75.2 (1.0)	76.1 (1.1)	75.8 (1.6)	76.3 (1.5)	-0.285	-0.343	0.5
		30-64	66.7 (1.6)	65.2 (1.6)	63.4 (1.6)	65.7 (1.9)	66.4 (2.7)	64.8 (2.5)	-0.504^*^	-0.604^*^	-1.6
		≥65	85.3 (1.2)	85.4 (1.0)	86.2 (1.0)	85.0 (1.1)	84.1 (1.4)	85.8 (1.6)	-0.009	-0.030	1.8
Treatment	Total (yr)										
		Total (≥30)	60.7 (1.0)	63.6 (0.9)	65.3 (0.9)	66.8 (1.0)	67.1 (1.4)	66.5 (1.4)	0.326^*^	0.327^*^	-0.6
		30-64	49.0 (1.3)	51.7 (1.3)	53.7 (1.1)	55.9 (1.3)	56.4 (2.0)	55.4 (1.8)	0.462^*^	0.466^*^	-1.1
		≥65	80.9 (1.1)	82.5 (0.9)	83.1 (0.9)	82.3 (0.9)	81.6 (1.2)	83.0 (1.5)	0.185	0.197	1.4
	Men (yr)										
		Total (≥30)	51.4 (1.4)	55.4 (1.4)	59.7 (1.2)	61.7 (1.4)	61.9 (1.9)	61.4 (2.0)	0.785^***^	0.800^*^	-0.5
		30-64	42.2 (1.7)	45.6 (1.7)	50.5 (1.5)	52.6 (1.7)	52.3 (2.5)	52.8 (2.4)	0.907^*^	0.918^*^	0.5
		≥65	78.2 (1.6)	81.0 (1.4)	82.3 (1.2)	82.1 (1.5)	81.8 (2.0)	82.3 (2.3)	0.514^*^	0.577^*^	0.5
	Women (yr)										
		Total (≥30)	70.7 (1.1)	72.7 (1.0)	72.0 (1.0)	72.8 (1.2)	72.7 (1.7)	72.9 (1.6)	-0.157	-0.174	0.2
		30-64	59.7 (1.6)	61.7 (1.7)	59.4 (1.7)	61.6 (1.9)	62.8 (2.8)	60.3 (2.6)	-0.238	-0.254	-2.4
		≥65	82.5 (1.3)	83.5 (1.1)	83.7 (1.1)	82.4 (1.2)	81.4 (1.6)	83.4 (1.7)	-0.007	-0.022	1.9
Control among treated persons	Total (yr)										
		Total (≥30)	69.1 (1.0)	72.0 (1.0)	73.1 (0.9)	72.2 (1.0)	72.0 (1.4)	72.4 (1.4)	0.408^*^	0.499^*^	0.4
		30-64	70.2 (1.5)	72.1 (1.5)	74.3 (1.4)	75.0 (1.4)	75.0 (2.1)	75.0 (2.0)	0.592^*^	0.644^*^	0.0
		≥65	67.9 (1.3)	72.0 (1.3)	72.0 (1.2)	69.4 (1.4)	69.1 (2.0)	69.8 (1.9)	0.236	0.376	0.6
	Men (yr)										
		Total (≥30)	71.8 (1.5)	72.4 (1.4)	75.8 (1.3)	75.3 (1.4)	76.4 (1.9)	74.2 (2.1)	0.456^*^	0.607^*^	-2.2
		30-64	71.6 (2.0)	69.6 (2.1)	73.7 (1.8)	74.0 (2.0)	74.4 (3.0)	73.6 (2.8)	0.388	0.434	-0.7
		≥65	72.1 (2.1)	76.4 (2.0)	79.0 (1.6)	77.1 (2.0)	79.1 (2.5)	75.1 (3.2)	0.571	0.897^*^	-4.0
	Women (yr)										
		Total (≥30)	67.0 (1.3)	71.7 (1.3)	70.5 (1.2)	69.1 (1.4)	68.0 (1.9)	70.4 (1.9)	0.332	0.361	2.5
		30-64	68.6 (2.1)	75.1 (1.9)	75.3 (1.8)	76.6 (2.1)	75.9 (2.9)	77.5 (3.0)	0.895^*^	0.943^*^	1.6
		≥65	65.7 (1.6)	69.3 (1.6)	67.3 (1.5)	64.4 (1.8)	62.6 (2.6)	66.2 (2.4)	-0.072	-0.055	3.6

Values are presented as the crude weighted % (standard error).

* p<0.05,

*** p<0.001.

**Table 3.** Trends in the prevalence and management of diabetes mellitus among Korean men and women aged 30 years or older in the 2011-2020 Korea National Health and Nutrition Examination Survey

Indicators	Variables		2011-2012	2013-2015	2016-2018	2019-2020	2019	2020	Trend		Difference 2019 to 2020
									2011-2020 (β estimate)	2011-2019 (β estimate)	
Prevalence	Total (yr)										
		Total (≥30)	12.1 (0.4)	12.9 (0.4)	13.9 (0.4)	15.6 (0.5)	14.5 (0.6)	16.7 (0.7)	0.125	-0.002	2.2^*^
		30-64	9.6 (0.5)	9.9 (0.4)	10.4 (0.4)	11.8 (0.5)	10.7 (0.6)	13.0 (0.7)	0.099	-0.032	2.3^*^
		≥65	25.3 (1.0)	28.2 (1.0)	28.4 (0.8)	29.6 (0.9)	29.0 (1.2)	30.1 (1.3)	0.265	0.175	1.0
	Men (yr)										
		Total (≥30)	13.5 (0.7)	14.6 (0.6)	15.6 (0.5)	17.7 (0.7)	16.3 (0.9)	19.2 (1.0)	0.224^*^	0.067	2.9^*^
		30-64	11.5 (0.7)	12.3 (0.6)	13.0 (0.5)	14.9 (0.8)	13.2 (0.9)	16.6 (1.2)	0.202	0.022	3.4^*^
		≥65	26.0 (1.5)	27.4 (1.4)	28.4 (1.2)	30.0 (1.5)	30.1 (2.1)	29.8 (2.1)	0.380	0.356	-0.3
	Women (yr)										
		Total (≥30)	10.8 (0.5)	11.4 (0.4)	12.2 (0.4)	13.5 (0.5)	12.7 (0.7)	14.3 (0.8)	0.016	-0.080	1.6
		30-64	7.6 (0.5)	7.5 (0.4)	7.7 (0.4)	8.6 (0.5)	8.0 (0.7)	9.2 (0.8)	-0.016	-0.098	1.2
		≥65	24.8 (1.3)	28.9 (1.4)	28.5 (1.1)	29.3 (1.1)	28.2 (1.5)	30.2 (1.5)	0.165	0.018	2.0
Awareness	Total (yr)										
		Total (≥30)	60.8 (1.7)	61.0 (1.4)	65.0 (1.2)	65.8 (1.4)	65.2 (1.9)	66.3 (2.0)	0.374	0.295	1.1
		30-64	55.1 (2.3)	54.3 (1.9)	56.6 (1.7)	58.6 (1.9)	57.9 (2.7)	59.1 (2.7)	0.224	0.026	1.2
		≥65	72.1 (2.2)	72.6 (2.0)	77.9 (1.4)	76.4 (1.5)	75.4 (2.2)	77.3 (2.3)	0.739^*^	0.871^*^	1.9
	Men (yr)										
		Total (≥30)	59.7 (2.6)	59.5 (2.1)	61.2 (1.6)	62.6 (1.9)	63.3 (2.6)	62.1 (2.8)	0.158	0.101	-1.2
		30-64	54.8 (3.2)	54.1 (2.5)	54.6 (2.1)	56.6 (2.5)	57.1 (3.5)	56.2 (3.6)	0.047	-0.099	-0.9
		≥65	73.1 (3.4)	73.0 (2.9)	76.0 (2.1)	75.6 (2.5)	75.6 (3.1)	75.7 (3.9)	0.663	0.841	0.1
	Women (yr)										
		Total (≥30)	62.2 (2.3)	62.8 (1.8)	69.8 (1.6)	69.7 (1.8)	67.5 (2.8)	71.7 (2.1)	0.679^*^	0.577	4.2
		30-64	55.5 (3.5)	54.7 (2.7)	60.0 (2.5)	62.1 (2.9)	59.2 (4.3)	64.5 (3.8)	0.585	0.308	5.3
		≥65	71.4 (2.9)	72.3 (2.4)	79.4 (1.8)	77.0 (2.1)	75.3 (3.1)	78.5 (2.8)	0.804^*^	0.910	3.2
Treatment	Total (yr)										
		Total (≥30)	54.3 (1.7)	54.8 (1.4)	60.1 (1.3)	61.4 (1.5)	60.8 (2.0)	61.9 (2.2)	0.667^*^	0.610^*^	1.1
		30-64	48.4 (2.3)	47.9 (1.9)	51.8 (1.7)	53.4 (2.1)	52.4 (2.7)	54.2 (3.0)	0.463	0.303	1.7
		≥65	65.9 (2.1)	66.9 (2.0)	72.9 (1.5)	73.3 (1.6)	72.6 (2.2)	74.0 (2.4)	1.149^*^	1.279^*^	1.4
	Men (yr)										
		Total (≥30)	52.9 (2.5)	52.8 (2.1)	56.6 (1.7)	57.1 (2.1)	57.9 (2.8)	56.5 (3.1)	0.398	0.424	-1.3
		30-64	47.7 (3.1)	47.2 (2.5)	50.4 (2.1)	50.7 (2.7)	50.9 (3.7)	50.5 (4.0)	0.289	0.238	-0.5
		≥65	67.0 (3.5)	66.9 (2.9)	70.6 (2.2)	71.2 (2.6)	71.6 (3.1)	70.7 (4.1)	0.922	1.130^*^	-0.9
	Women (yr)										
		Total (≥30)	56.1 (2.4)	57.3 (1.8)	64.6 (1.7)	66.8 (1.8)	64.5 (2.7)	68.9 (2.2)	1.052	0.891^*^	4.5
		30-64	49.5 (3.5)	49.0 (2.6)	54.3 (2.6)	58.3 (2.8)	55.0 (4.2)	61.0 (3.8)	0.834	0.492	6.0
		≥65	65.1 (2.9)	66.9 (2.5)	74.7 (2.0)	75.0 (2.1)	73.3 (3.0)	76.4 (2.8)	1.341^*^	1.414^*^	3.1
Control among treated persons	Total (yr)										
		Total (≥30)	23.3 (2.0)	22.3 (1.5)	25.8 (1.4)	25.2 (1.5)	25.5 (2.0)	25.0 (2.2)	0.263	0.346	-0.5
		30-64	19.9 (2.8)	19.6 (2.1)	22.1 (1.9)	20.6 (2.2)	19.5 (2.9)	21.5 (3.2)	0.154	0.172	2.0
		≥65	28.1 (2.8)	25.8 (2.0)	29.8 (1.9)	30.2 (2.0)	31.6 (2.7)	29.0 (2.9)	0.397	0.602	-2.6
	Men (yr)										
		Total (≥30)	20.6 (2.5)	23.2 (2.1)	25.1 (1.9)	23.8 (2.1)	25.9 (2.7)	21.9 (3.0)	0.277	0.612	-4.0
		30-64	20.2 (3.5)	19.4 (2.8)	22.1 (2.6)	17.5 (2.7)	19.0 (3.8)	16.4 (3.8)	-0.181	0.175	-2.6
		≥65	21.5 (3.5)	30.0 (3.2)	29.9 (2.7)	33.4 (3.1)	35.7 (4.0)	31.2 (4.6)	1.104^*^	1.470^*^	-4.5
	Women (yr)										
		Total (≥30)	26.3 (3.0)	21.3 (2.1)	26.5 (1.8)	26.7 (2.2)	24.9 (2.9)	28.2 (3.2)	0.272	0.046	3.3
		30-64	19.5 (4.1)	19.8 (3.2)	22.0 (2.8)	25.3 (3.5)	20.2 (4.4)	29.3 (5.2)	0.709	0.198	9.1
		≥65	33.3 (4.0)	22.7 (2.7)	29.7 (2.4)	27.8 (2.7)	28.3 (3.8)	27.4 (3.8)	-0.158	-0.089	-0.9

Values are presented as the crude weighted % (standard error).

* p<0.05.

**Table 4.** Trends in the prevalence and management of hypercholesterolemia among Korean men and women aged 30 years or older in the 2010-2020 Korea National Health and Nutrition Examination Survey

Indicators	Variables		2010-2012	2013-2015	2016-2018	2019-2020	2019	2020	Trend		Difference 2019 to 2020
									2010-2020 (β estimate)	2010-2019 (β estimate)	
Prevalence	Total (yr)										
		Total (≥30)	14.7 (0.4)	17.1 (0.4)	23.2 (0.4)	26.7 (0.6)	25.8 (0.8)	27.5 (0.9)	1.237^***^	1.215^***^	1.8
		30-64	13.4 (0.4)	15.0 (0.4)	20.8 (0.5)	23.1 (0.6)	22.3 (0.9)	23.9 (0.9)	1.011^***^	0.996^***^	1.5
		≥65	21.2 (0.9)	27.3 (0.9)	33.5 (0.8)	39.9 (1.1)	38.9 (1.5)	40.8 (1.6)	2.315^***^	2.279^***^	1.9
	Men (yr)										
		Total (≥30)	12.9 (0.5)	15.1 (0.5)	21.1 (0.6)	24.5 (0.8)	22.6 (1.1)	26.4 (1.2)	1.302^***^	1.193^***^	3.9^*^
		30-64	12.8 (0.6)	14.6 (0.6)	20.4 (0.7)	23.1 (0.9)	21.3 (1.2)	24.8 (1.3)	1.148^***^	1.060^***^	3.5^*^
		≥65	13.7 (1.1)	17.6 (1.2)	24.2 (1.1)	30.9 (1.6)	28.3 (2.0)	33.3 (2.5)	2.134^***^	1.919^***^	5.0
	Women (yr)										
		Total (≥30)	16.4 (0.5)	19.0 (0.5)	25.3 (0.6)	28.7 (0.8)	28.8 (1.1)	28.6 (1.1)	1.180^***^	1.240^***^	-0.2
		30-64	14.1 (0.6)	15.5 (0.6)	21.1 (0.6)	23.1 (0.8)	23.4 (1.2)	22.9 (1.1)	0.868^***^	0.928^***^	-0.5
		≥65	26.6 (1.2)	35.0 (1.3)	40.6 (1.2)	46.8 (1.5)	47.0 (2.3)	46.6 (2.0)	2.502^***^	2.603^***^	-0.4
Awareness	Total (yr)										
		Total (≥30)	47.4 (1.4)	57.7 (1.3)	60.1 (1.0)	64.6 (1.1)	61.7 (1.6)	67.2 (1.5)	1.276^***^	1.184^***^	5.5^*^
		30-64	42.4 (1.6)	50.3 (1.5)	51.8 (1.2)	55.6 (1.5)	53.0 (2.1)	58.1 (1.9)	1.019^***^	0.883^*^	5.1^*^
		≥65	64.3 (2.3)	77.9 (1.7)	81.7 (1.3)	83.9 (1.3)	81.0 (2.0)	86.5 (1.7)	2.149^***^	2.186^***^	5.5^*^
	Men (yr)										
		Total (≥30)	45.2 (2.3)	51.4 (2.0)	57.3 (1.5)	63.2 (1.8)	59.7 (2.7)	66.0 (2.3)	1.286^***^	1.117^*^	6.3
		30-64	41.5 (2.5)	45.8 (2.3)	51.3 (1.7)	56.6 (2.1)	54.1 (3.2)	58.8 (2.7)	1.137^*^	0.990^*^	4.7
		≥65	66.9 (4.1)	77.5 (3.2)	81.8 (2.0)	84.4 (2.2)	78.9 (3.9)	88.7 (2.6)	2.040^***^	1.776^*^	9.9^*^
	Women (yr)										
		Total (≥30)	49.1 (1.7)	62.4 (1.6)	62.4 (1.2)	65.8 (1.4)	63.2 (1.8)	68.3 (2.1)	1.211^***^	1.208^***^	5.1
		30-64	43.2 (2.1)	54.5 (2.0)	52.2 (1.5)	54.6 (1.9)	51.9 (2.4)	57.3 (2.8)	0.826^*^	0.740^*^	5.4
		≥65	63.4 (2.7)	78.0 (2.0)	81.7 (1.5)	83.7 (1.6)	82.0 (2.4)	85.3 (2.1)	2.200^***^	2.372^***^	3.4
Treatment	Total (yr)										
		Total (≥30)	37.3 (1.4)	45.5 (1.3)	50.3 (1.0)	56.7 (1.2)	53.1 (1.6)	60.0 (1.6)	1.604^***^	1.396^***^	6.9^*^
		30-64	31.6 (1.5)	36.6 (1.4)	40.7 (1.2)	47.0 (1.5)	43.5 (2.0)	50.4 (2.0)	1.412^***^	1.126^***^	6.9^*^
		≥65	56.3 (2.6)	69.9 (1.8)	75.2 (1.3)	77.5 (1.5)	74.3 (2.1)	80.3 (2.0)	2.289^***^	2.301^***^	6.0^*^
	Men (yr)										
		Total (≥30)	35.8 (2.0)	39.8 (2.0)	48.8 (1.5)	56.1 (1.8)	51.0 (2.5)	60.3 (2.4)	1.679^***^	1.362^***^	9.4^*^
		30-64	31.2 (2.2)	33.3 (2.2)	41.8 (1.8)	48.9 (2.1)	44.8 (2.9)	52.5 (3.0)	1.602^***^	1.299^*^	7.7
		≥65	62.6 (4.2)	69.9 (3.6)	77.9 (2.1)	79.2 (2.4)	72.0 (3.8)	84.9 (3.0)	2.082^***^	1.681^*^	12.9^*^
	Women (yr)										
		Total (≥30)	38.4 (1.8)	49.9 (1.6)	51.5 (1.2)	57.2 (1.4)	54.7 (1.8)	59.7 (2.1)	1.455^***^	1.372^***^	5.0
		30-64	31.9 (1.9)	39.7 (1.9)	39.6 (1.5)	45.1 (1.9)	42.3 (2.4)	48.0 (2.8)	1.113^***^	0.895^*^	5.8
		≥65	54.0 (2.9)	69.9 (2.1)	73.9 (1.6)	76.6 (1.9)	75.4 (2.6)	77.8 (2.6)	2.339^***^	2.535^***^	2.4
Control among treated persons	Total (yr)										
		Total (≥30)	78.8 (1.6)	84.3 (1.2)	84.0 (0.9)	85.1 (1.0)	82.8 (1.5)	86.9 (1.3)	0.541^*^	0.396	4.1^*^
		30-64	77.5 (2.2)	83.1 (1.7)	82.5 (1.4)	83.5 (1.4)	79.1 (2.2)	87.0 (1.8)	0.583^*^	0.279	8.0^*^
		≥65	81.3 (2.1)	85.9 (1.5)	86.1 (1.1)	87.1 (1.4)	87.6 (2.1)	86.7 (1.8)	0.484	0.584	-0.9
	Men (yr)										
		Total (≥30)	79.3 (2.8)	88.4 (1.8)	85.7 (1.5)	85.0 (1.5)	83.2 (2.2)	86.2 (2.1)	0.220	0.232	3.0
		30-64	77.0 (3.5)	86.0 (2.4)	83.8 (2.0)	83.4 (2.0)	81.3 (3.0)	84.9 (2.8)	0.398	0.383	3.6
		≥65	85.8 (3.4)	93.6 (1.8)	89.8 (1.8)	88.1 (2.2)	87.2 (3.4)	88.6 (3.0)	-0.146	-0.146	1.4
	Women (yr)										
		Total (≥30)	78.5 (1.9)	81.8 (1.6)	82.7 (1.2)	85.1 (1.3)	82.5 (2.1)	87.5 (1.5)	0.724^*^	0.473	5.0
		30-64	78.0 (2.6)	80.8 (2.4)	81.1 (1.9)	83.5 (1.9)	76.8 (3.1)	89.5 (2.2)	0.763^*^	0.191	12.7^*^
		≥65	79.3 (2.6)	82.9 (2.0)	84.3 (1.5)	86.6 (1.7)	87.8 (2.8)	85.6 (2.2)	0.714^*^	0.846^*^	-2.2

Values are presented as the crude weighted % (standard error).

* p<0.05,

*** p<0.001.

## SUPPLEMENTARY MATERIALS

Supplementary Material 1. Trends in risk factors among Korean men and women with hypertension in the 2010-2020 Korea National Health and Nutrition Examination Survey

Supplementary Material 2. Trends in risk factors among Korean men and women with diabetes mellitus in the 2011-2020 Korea National Health and Nutrition Examination Survey

Supplementary Material 3. Trends in risk factors among Korean men and women with hypercholesterolemia in the 2010-2020 Korea National Health and Nutrition Examination Survey
