# Supplementary material for: Changes in the management of hypertension, diabetes mellitus, and hypercholesterolemia in Korean adults before and during the COVID-19 pandemic: data from the 2010-2020 Korea National Health and Nutrition Examination Survey
Source: Epidemiol Health. 2023 Feb 1;45:e2023014. doi: 10.4178/epih.e2023014 (PMC10581891; doi:10.4178/epih.e2023014)
Supplement: Supplementary Material 1. — Trends in risk factors among Korean men and women with hypertension in the 2010-2020 Korea National Health and Nutrition Examination Survey [file epih-45-e2023014-Supplementary-1.docx]

**Supplementary Material 1. Trends in risk factors among Korean men and women with hypertension in the 2010-2020 Korea National Health and Nutrition Examination Survey**

| **Indicators** | **Variables** | **2010-2012** | | **2013-2015** | | **2016-2018** | |  | |  | | | | **Trend** | | **Difference**  **2019 to 2020** |
| --- | --- | --- | --- | --- | --- | --- | --- | --- | --- | --- | --- | --- | --- | --- | --- | --- |
|  |  |  |  |  |  |  |  | **2019-2020** | | **2019** | | **2020** | | **2010-2020**  **(β estimate)** | **2010-2019**  **(β estimate)** |  |
| Obesity | Total |  |  |  |  |  |  |  |  |  |  |  |  |  |  |  |
|  | ≥30 | 47.3 | (0.9) | 50.1 | (0.9) | 50.8 | (0.8) | 51.5 | (1.0) | 48.4 | (1.4) | 54.6 | (1.5) | 0.642*** | 0.488* | 6.1* |
|  | 30-64 | 51.9 | (1.2) | 55.3 | (1.2) | 55.3 | (1.0) | 58.1 | (1.4) | 54.1 | (1.8) | 61.8 | (1.9) | 0.714*** | 0.430* | 7.7* |
|  | ≥65 | 39.4 | (1.2) | 41.7 | (1.2) | 43.7 | (1.1) | 41.9 | (1.3) | 40.6 | (1.8) | 43.3 | (1.9) | 0.557* | 0.615* | 2.7 |
|  | Men |  |  |  |  |  |  |  |  |  |  |  |  |  |  |  |
|  | ≥30 | 48.6 | (1.3) | 52.8 | (1.2) | 53.2 | (1.1) | 56.6 | (1.4) | 52.4 | (1.9) | 60.4 | (1.9) | 1.100*** | 0.847*** | 8.0* |
|  | 30-64 | 54.1 | (1.6) | 58.5 | (1.5) | 59.2 | (1.3) | 63.0 | (1.7) | 58.1 | (2.3) | 67.1 | (2.3) | 1.077*** | 0.745* | 9.0* |
|  | ≥65 | 32.6 | (1.8) | 38.1 | (1.9) | 38.5 | (1.6) | 42.0 | (2.0) | 40.3 | (2.7) | 43.6 | (3.1) | 1.189*** | 1.136*** | 3.3 |
|  | Women |  |  |  |  |  |  |  |  |  |  |  |  |  |  |  |
|  | ≥30 | 46.0 | (1.1) | 47.0 | (1.1) | 47.8 | (1.1) | 45.5 | (1.3) | 44.1 | (1.8) | 46.9 | (2.0) | 0.122 | 0.110 | 2.8 |
|  | 30-64 | 48.6 | (1.6) | 50.0 | (1.8) | 48.4 | (1.7) | 49.4 | (2.2) | 47.9 | (2.7) | 51.2 | (3.3) | 0.064 | -0.098 | 3.3 |
|  | ≥65 | 43.3 | (1.5) | 44.0 | (1.5) | 47.2 | (1.4) | 41.9 | (1.6) | 40.8 | (2.2) | 43.1 | (2.5) | 0.245 | 0.386 | 2.3 |
| Current cigarette smoking | Total |  |  |  |  |  |  |  |  |  |  |  |  |  |  |  |
|  | ≥30 | 23.0 | (0.8) | 21.5 | (0.8) | 20.6 | (0.7) | 18.6 | (0.8) | 17.7 | (1.0) | 19.6 | (1.2) | -0.260* | -0.289* | 1.9 |
|  | 30-64 | 29.6 | (1.1) | 28.8 | (1.2) | 28.3 | (1.1) | 25.5 | (1.1) | 24.0 | (1.5) | 26.9 | (1.7) | -0.279 | -0.309 | 2.9 |
|  | ≥65 | 11.3 | (0.7) | 9.4 | (0.7) | 8.5 | (0.7) | 8.8 | (0.8) | 9.1 | (1.1) | 8.6 | (1.2) | -0.250* | -0.281* | -0.5 |
|  | Men |  |  |  |  |  |  |  |  |  |  |  |  |  |  |  |
|  | ≥30 | 40.1 | (1.2) | 36.6 | (1.3) | 34.0 | (1.1) | 31.7 | (1.3) | 31.1 | (1.7) | 32.3 | (1.8) | -0.750*** | -0.785*** | 1.2 |
|  | 30-64 | 45.4 | (1.5) | 43.0 | (1.6) | 40.4 | (1.5) | 37.6 | (1.6) | 37.1 | (2.3) | 38.1 | (2.3) | -0.799*** | -0.823* | 1.0 |
|  | ≥65 | 24.6 | (1.5) | 20.1 | (1.6) | 18.2 | (1.4) | 18.6 | (1.7) | 19.0 | (2.2) | 18.1 | (2.7) | -0.620* | -0.671* | -0.9 |
|  | Women |  |  |  |  |  |  |  |  |  |  |  |  |  |  |  |
|  | ≥30 | 4.5 | (0.5) | 4.1 | (0.5) | 4.5 | (0.5) | 3.3 | (0.5) | 3.3 | (0.7) | 3.4 | (0.7) | -0.018 | 0.007 | 0.0 |
|  | 30-64 | 5.3 | (0.8) | 5.6 | (0.9) | 7.1 | (0.9) | 4.6 | (0.9) | 4.3 | (1.2) | 5.0 | (1.3) | 0.164 | 0.234 | 0.7 |
|  | ≥65 | 3.7 | (0.6) | 2.6 | (0.5) | 2.1 | (0.4) | 2.2 | (0.5) | 2.4 | (0.7) | 2.0 | (0.8) | -0.210* | -0.241* | -0.4 |
| High-risk alcohol drinking | Total |  |  |  |  |  |  |  |  |  |  |  |  |  |  |  |
|  | ≥30 | 15.0 | (0.7) | 15.6 | (0.7) | 17.1 | (0.6) | 15.8 | (0.7) | 14.7 | (1.0) | 16.7 | (1.1) | 0.400*** | 0.435*** | 2.0 |
|  | 30-64 | 21.4 | (1.0) | 22.9 | (1.1) | 24.8 | (1.0) | 22.1 | (1.1) | 19.6 | (1.6) | 24.4 | (1.7) | 0.363* | 0.355 | 4.7 |
|  | ≥65 | 3.7 | (0.4) | 3.5 | (0.4) | 5.2 | (0.5) | 6.7 | (0.7) | 8.1 | (1.1) | 5.3 | (0.8) | 0.435*** | 0.532*** | -2.8* |
|  | Men |  |  |  |  |  |  |  |  |  |  |  |  |  |  |  |
|  | ≥30 | 26.5 | (1.2) | 25.7 | (1.2) | 27.2 | (1.0) | 26.2 | (1.2) | 25.0 | (1.8) | 27.3 | (1.6) | 0.331 | 0.344 | 2.3 |
|  | 30-64 | 32.5 | (1.6) | 32.7 | (1.5) | 33.4 | (1.3) | 30.9 | (1.6) | 28.2 | (2.5) | 33.2 | (2.2) | 0.101 | 0.051 | 5.0 |
|  | ≥65 | 9.1 | (1.1) | 7.4 | (1.0) | 11.8 | (1.1) | 15.7 | (1.5) | 18.5 | (2.3) | 12.9 | (1.9) | 0.922*** | 1.092*** | -5.6 |
|  | Women |  |  |  |  |  |  |  |  |  |  |  |  |  |  |  |
|  | ≥30 | 2.5 | (0.4) | 3.9 | (0.5) | 5.0 | (0.5) | 3.5 | (0.5) | 3.8 | (0.7) | 3.3 | (0.6) | 0.280*** | 0.375*** | -0.5 |
|  | 30-64 | 4.2 | (0.7) | 6.8 | (0.9) | 9.7 | (1.0) | 6.8 | (0.9) | 6.6 | (1.3) | 7.0 | (1.3) | 0.525*** | 0.651*** | 0.4 |
|  | ≥65 | 0.5 | (0.2) | 1.0 | (0.4) | 0.7 | (0.2) | 0.7 | (0.3) | 1.2 | (0.6) | 0.1 | (0.1) | 0.027 | 0.080 | -1.1 |
| Aerobic physical activity | Total |  |  |  |  |  |  |  |  |  |  |  |  |  |  |  |
|  | ≥30 | - | - | 44.5 | (1.3) | 37.6 | (0.8) | 36.6 | (1.0) | 36.5 | (1.3) | 36.8 | (1.4) | -1.612*** | -2.024*** | 0.4 |
|  | 30-64 | - | - | 50.3 | (1.6) | 43.8 | (1.1) | 40.5 | (1.4) | 39.7 | (1.9) | 41.2 | (2.0) | -1.871*** | -2.331*** | 1.6 |
|  | ≥65 | - | - | 35.5 | (1.5) | 27.9 | (1.0) | 30.7 | (1.3) | 31.9 | (1.8) | 29.3 | (1.9) | -1.192*** | -1.490* | -2.5 |
|  | Men |  |  |  |  |  |  |  |  |  |  |  |  |  |  |  |
|  | ≥30 | - | - | 50.9 | (1.7) | 42.6 | (1.1) | 40.1 | (1.4) | 39.1 | (1.8) | 40.9 | (2.1) | -2.150*** | -2.849*** | 1.8 |
|  | 30-64 | - | - | 52.7 | (2.1) | 45.6 | (1.4) | 41.5 | (1.8) | 40.5 | (2.4) | 42.4 | (2.7) | -2.117*** | -2.678*** | 1.9 |
|  | ≥65 | - | - | 46.5 | (2.4) | 35.1 | (1.6) | 36.7 | (2.0) | 36.4 | (2.5) | 37.1 | (3.1) | -2.205*** | -3.187*** | 0.8 |
|  | Women |  |  |  |  |  |  |  |  |  |  |  |  |  |  |  |
|  | ≥30 | - | - | 37.3 | (1.5) | 31.6 | (1.1) | 32.5 | (1.3) | 33.5 | (1.8) | 31.4 | (2.0) | -1.063* | -1.165* | -2.1 |
|  | 30-64 | - | - | 46.3 | (2.3) | 40.6 | (1.7) | 38.7 | (2.1) | 38.4 | (2.8) | 39.0 | (3.2) | -1.529* | -1.800* | 0.6 |
|  | ≥65 | - | - | 28.6 | (1.7) | 23.1 | (1.2) | 26.4 | (1.6) | 28.7 | (2.3) | 23.7 | (2.2) | -0.605 | -0.472 | -5.1 |
| Perceived Stress | Total |  |  |  |  |  |  |  |  |  |  |  |  |  |  |  |
|  | ≥30 | 25.3 | (0.8) | 23.5 | (0.8) | 23.8 | (0.6) | 24.8 | (0.9) | 23.2 | (1.2) | 26.2 | (1.4) | 0.072 | -0.080 | 3.0 |
|  | 30-64 | 26.7 | (1.0) | 26.1 | (1.1) | 27.0 | (0.9) | 29.9 | (1.3) | 27.1 | (1.7) | 32.6 | (2.0) | 0.473* | 0.213 | 5.5* |
|  | ≥65 | 22.9 | (1.1) | 19.2 | (1.0) | 18.9 | (0.8) | 17.4 | (1.0) | 18.0 | (1.4) | 16.7 | (1.3) | -0.577*** | -0.561* | -1.3 |
|  | Men |  |  |  |  |  |  |  |  |  |  |  |  |  |  |  |
|  | ≥30 | 22.2 | (1.1) | 23.1 | (1.1) | 22.7 | (0.8) | 25.3 | (1.2) | 23.2 | (1.8) | 27.2 | (1.7) | 0.509* | 0.352 | 4.0 |
|  | 30-64 | 25.5 | (1.4) | 27.8 | (1.5) | 26.7 | (1.1) | 31.5 | (1.7) | 28.9 | (2.4) | 33.7 | (2.2) | 0.706* | 0.467 | 4.8 |
|  | ≥65 | 12.6 | (1.2) | 11.1 | (1.3) | 12.9 | (1.0) | 11.5 | (1.2) | 11.6 | (1.6) | 11.3 | (1.7) | -0.024 | 0.039 | -0.3 |
|  | Women |  |  |  |  |  |  |  |  |  |  |  |  |  |  |  |
|  | ≥30 | 28.6 | (1.1) | 23.9 | (1.0) | 25.2 | (0.9) | 24.1 | (1.1) | 23.2 | (1.4) | 25.1 | (1.7) | -0.374* | -0.503* | 1.8 |
|  | 30-64 | 28.5 | (1.7) | 23.5 | (1.5) | 27.5 | (1.4) | 27.2 | (1.8) | 24.3 | (2.1) | 30.4 | (3.0) | 0.076 | -0.184 | 6.2 |
|  | ≥65 | 28.8 | (1.4) | 24.4 | (1.4) | 23.0 | (1.2) | 21.4 | (1.3) | 22.3 | (1.9) | 20.5 | (1.7) | -0.787*** | -0.790* | -1.8 |
| Excessive sodium intake | Total |  |  |  |  |  |  |  |  |  |  |  |  |  |  |  |
|  | ≥30 | 84.3 | (0.6) | 75.7 | (0.8) | 70.3 | (0.8) | 70.9 | (0.9) | 70.5 | (1.2) | 71.3 | (1.3) | -1.417*** | -1.668*** | 1.7 |
|  | 30-64 | 90.8 | (0.6) | 83.0 | (1.0) | 78.1 | (1.0) | 77.9 | (1.1) | 77.9 | (1.5) | 77.9 | (1.8) | -1.546*** | -1.743*** | 0.5 |
|  | ≥65 | 73.1 | (1.2) | 64.9 | (1.2) | 59.3 | (1.2) | 62.2 | (1.3) | 61.7 | (1.7) | 62.7 | (1.9) | -1.152*** | -1.472*** | 2.3 |
|  | Men |  |  |  |  |  |  |  |  |  |  |  |  |  |  |  |
|  | ≥30 | 92.9 | (0.6) | 87.7 | (0.8) | 82.6 | (0.9) | 82.9 | (1.0) | 83.4 | (1.3) | 82.4 | (1.6) | -1.085*** | -1.213*** | -0.8 |
|  | 30-64 | 95.8 | (0.6) | 92.0 | (1.0) | 87.0 | (1.1) | 85.9 | (1.3) | 87.6 | (1.4) | 84.5 | (2.1) | -1.181*** | -1.183*** | -3.2 |
|  | ≥65 | 84.4 | (1.5) | 78.3 | (1.5) | 73.4 | (1.5) | 77.4 | (1.6) | 76.3 | (2.2) | 78.5 | (2.4) | -0.731* | -1.137*** | 2.9 |
|  | Women |  |  |  |  |  |  |  |  |  |  |  |  |  |  |  |
|  | ≥30 | 75.0 | (1.0) | 62.2 | (1.2) | 55.2 | (1.1) | 57.2 | (1.3) | 56.9 | (1.7) | 57.4 | (1.8) | -2.037*** | -2.401*** | 1.9 |
|  | 30-64 | 83.0 | (1.3) | 68.9 | (1.9) | 62.5 | (1.7) | 64.9 | (2.1) | 63.9 | (2.8) | 66.0 | (3.2) | -2.318*** | -2.761*** | 2.5 |
|  | ≥65 | 66.6 | (1.6) | 55.6 | (1.5) | 48.7 | (1.5) | 50.8 | (1.6) | 51.0 | (2.1) | 50.6 | (2.2) | -1.720*** | -1.997*** | 1.9 |
| Excessive energy and fat intake | Total |  |  |  |  |  |  |  |  |  |  |  |  |  |  |  |
|  | ≥30 | 2.6 | (0.3) | 3.4 | (0.4) | 3.9 | (0.4) | 5.6 | (0.6) | 4.0 | (0.6) | 7.2 | (0.9) | 0.365*** | 0.220*** | 2.7* |
|  | 30-64 | 3.6 | (0.5) | 4.8 | (0.6) | 5.7 | (0.6) | 8.3 | (0.9) | 6.0 | (1.0) | 10.5 | (1.5) | 0.497*** | 0.298* | 3.7* |
|  | ≥65 | 0.9 | (0.2) | 1.3 | (0.3) | 1.3 | (0.2) | 2.3 | (0.5) | 1.6 | (0.5) | 2.9 | (0.8) | 0.160* | 0.095 | 1.0 |
|  | Men |  |  |  |  |  |  |  |  |  |  |  |  |  |  |  |
|  | ≥30 | 4.3 | (0.6) | 5.0 | (0.6) | 5.7 | (0.7) | 8.5 | (1.0) | 6.1 | (1.1) | 10.7 | (1.6) | 0.531*** | 0.297* | 4.1* |
|  | 30-64 | 5.1 | (0.8) | 6.7 | (0.8) | 7.7 | (0.9) | 11.2 | (1.4) | 8.4 | (1.6) | 13.7 | (2.1) | 0.659*** | 0.397* | 4.4 |
|  | ≥65 | 1.8 | (0.5) | 1.1 | (0.4) | 1.6 | (0.4) | 3.7 | (1.0) | 2.3 | (0.9) | 5.0 | (1.7) | 0.243* | 0.070 | 2.7 |
|  | Women |  |  |  |  |  |  |  |  |  |  |  |  |  |  |  |
|  | ≥30 | 0.8 | (0.2) | 1.7 | (0.3) | 1.5 | (0.3) | 2.3 | (0.5) | 1.8 | (0.6) | 2.8 | (0.8) | 0.158* | 0.116* | 0.6 |
|  | 30-64 | 1.3 | (0.3) | 1.9 | (0.5) | 2.2 | (0.5) | 3.5 | (0.9) | 2.5 | (1.1) | 4.6 | (1.6) | 0.218* | 0.123 | 1.5 |
|  | ≥65 | 0.4 | (0.2) | 1.5 | (0.4) | 1.0 | (0.3) | 1.2 | (0.4) | 1.1 | (0.5) | 1.4 | (0.6) | 0.099* | 0.106* | -0.1 |

Values are presented as weighted % (standard error) adjusted for age and household income level.

* p<0.05, *** p<0.001

Obesity: percentage of adults who have body mass index(BMI)≥25 kg/m^2^.

Current cigarette smoking: percentage of adults who have smoked at least 100 cigarettes during their lifetime and who are currently smokers.

High-risk alcohol drinking: percentage of adults who drink at least twice a week, with an average consumption of 7 drinks or more for men and 5 drinks or more for women.

Aerobic physical activity (PA): percentage of adults who have performed 150 minutes of moderate-intensity or 75 minutes of vigorous-intensity PA or an equivalent combination of moderate- and vigorous-intensity PA in a typical week (introduced in 2014 Korea National Health and Nutrition Examination Survey).

Perceived stress: percentage of adults who feel extremely or very stressed in their average daily life.

Excessive sodium intake: percentage of adults who consumed ≥ sodium of Intake Goal (Dietary Reference Intakes for Koreans).

Excessive energy and fat intake: percentage of adults who consumed ≥125% energy of the Estimated Energy Requirement and > fat intake of the Acceptable Macronutrient Distribution Ranges (Dietary Reference Intakes for Koreans).
