# Supplementary material for: Changes in the management of hypertension, diabetes mellitus, and hypercholesterolemia in Korean adults before and during the COVID-19 pandemic: data from the 2010-2020 Korea National Health and Nutrition Examination Survey
Source: Epidemiol Health. 2023 Feb 1;45:e2023014. doi: 10.4178/epih.e2023014 (PMC10581891; doi:10.4178/epih.e2023014)
Supplement: Supplementary Material 2. — Trends in risk factors among Korean men and women with diabetes mellitus in the 2011-2020 Korea National Health and Nutrition Examination Survey [file epih-45-e2023014-Supplementary-2.docx]

**Supplementary Material 2. Trends in risk factors among Korean men and women with diabetes mellitus in the 2011-2020 Korea National Health and Nutrition Examination Survey**

| **Indicators** | **Variables** | **2011-2012** | | **2013-2015** | | **2016-2018** | |  | |  | | | | **Trend** | | **Difference**  **2019 to 2020** |
| --- | --- | --- | --- | --- | --- | --- | --- | --- | --- | --- | --- | --- | --- | --- | --- | --- |
|  |  |  |  |  |  |  |  | **2019-2020** | | **2019** | | **2020** | | **2011-2020**  **(β estimate)** | **2011-2019**  **(β estimate)** |  |
| Obesity | Total |  |  |  |  |  |  |  |  |  |  |  |  |  |  |  |
|  | ≥30 | 49.5 | (1.8) | 48.6 | (1.3) | 53.2 | (1.2) | 54.3 | (1.4) | 54.5 | (1.9) | 54.2 | (2.0) | 0.863*** | 0.913* | -0.2 |
|  | 30-64 | 54.2 | (2.4) | 51.6 | (1.8) | 56.4 | (1.6) | 61.1 | (2.0) | 62.8 | (2.7) | 59.7 | (2.8) | 1.040* | 1.043* | -3.1 |
|  | ≥65 | 40.3 | (2.4) | 43.3 | (1.9) | 48.2 | (1.7) | 44.0 | (1.8) | 42.5 | (2.4) | 45.5 | (2.6) | 0.554 | 0.684 | 2.9 |
|  | Men |  |  |  |  |  |  |  |  |  |  |  |  |  |  |  |
|  | ≥30 | 42.8 | (2.4) | 48.1 | (2.0) | 51.0 | (1.7) | 56.6 | (1.9) | 56.9 | (2.6) | 56.3 | (2.8) | 1.664*** | 1.699*** | -0.6 |
|  | 30-64 | 47.0 | (3.2) | 51.5 | (2.5) | 55.3 | (2.2) | 63.3 | (2.5) | 64.8 | (3.4) | 62.0 | (3.4) | 1.829*** | 1.837*** | -2.7 |
|  | ≥65 | 31.3 | (3.2) | 39.5 | (2.7) | 41.1 | (2.4) | 42.1 | (2.8) | 41.3 | (3.6) | 42.7 | (4.3) | 1.247* | 1.370* | 1.4 |
|  | Women |  |  |  |  |  |  |  |  |  |  |  |  |  |  |  |
|  | ≥30 | 57.7 | (2.4) | 49.1 | (1.7) | 55.9 | (1.7) | 51.4 | (2.0) | 51.3 | (2.8) | 51.5 | (2.9) | -0.119 | -0.013 | 0.1 |
|  | 30-64 | 65.4 | (2.9) | 51.7 | (2.5) | 58.3 | (2.6) | 57.3 | (3.2) | 59.4 | (4.3) | 55.4 | (4.5) | -0.295 | -0.270 | -4.0 |
|  | ≥65 | 47.1 | (3.5) | 46.1 | (2.5) | 53.6 | (2.3) | 45.6 | (2.5) | 43.5 | (3.3) | 47.6 | (3.8) | 0.097 | 0.272 | 4.1 |
| Current cigarette smoking | Total |  |  |  |  |  |  |  |  |  |  |  |  |  |  |  |
|  | ≥30 | 26.0 | (1.5) | 24.9 | (1.3) | 21.1 | (1.1) | 22.3 | (1.3) | 22.2 | (1.6) | 22.5 | (2.0) | -0.375 | -0.496* | 0.3 |
|  | 30-64 | 31.3 | (2.1) | 32.3 | (1.8) | 28.4 | (1.5) | 29.9 | (1.9) | 29.8 | (2.4) | 30.1 | (2.8) | -0.343 | -0.466 | 0.3 |
|  | ≥65 | 15.7 | (1.6) | 11.8 | (1.3) | 9.8 | (1.0) | 11.0 | (1.3) | 11.3 | (1.7) | 10.6 | (1.9) | -0.448 | -0.576* | -0.7 |
|  | Men |  |  |  |  |  |  |  |  |  |  |  |  |  |  |  |
|  | ≥30 | 42.3 | (2.4) | 41.2 | (2.0) | 34.9 | (1.7) | 36.0 | (2.1) | 35.6 | (2.8) | 36.3 | (3.0) | -0.883* | -1.078* | 0.8 |
|  | 30-64 | 47.8 | (2.9) | 48.6 | (2.5) | 41.5 | (2.2) | 43.1 | (2.7) | 43.8 | (3.8) | 42.7 | (3.8) | -0.963* | -1.115* | -1.1 |
|  | ≥65 | 27.2 | (3.1) | 22.6 | (2.4) | 20.0 | (2.1) | 20.6 | (2.5) | 19.3 | (3.2) | 21.7 | (3.9) | -0.671 | -0.985 | 2.4 |
|  | Women |  |  |  |  |  |  |  |  |  |  |  |  |  |  |  |
|  | ≥30 | 6.5 | (1.1) | 4.8 | (0.9) | 3.7 | (0.7) | 5.0 | (0.9) | 5.7 | (1.3) | 4.4 | (1.3) | -0.136 | -0.165 | -1.4 |
|  | 30-64 | 6.0 | (1.6) | 5.9 | (1.3) | 5.5 | (1.2) | 6.7 | (1.5) | 6.6 | (2.2) | 6.8 | (2.2) | 0.135 | 0.082 | 0.2 |
|  | ≥65 | 7.0 | (1.7) | 3.6 | (1.1) | 1.8 | (0.6) | 3.3 | (0.8) | 4.9 | (1.4) | 2.0 | (0.8) | -0.448* | -0.464* | -2.9 |
| High-risk alcohol drinking | Total |  |  |  |  |  |  |  |  |  |  |  |  |  |  |  |
|  | ≥30 | 12.1 | (1.3) | 13.7 | (1.0) | 14.8 | (1.0) | 14.7 | (1.0) | 15.8 | (1.5) | 13.8 | (1.3) | 0.490* | 0.695*** | -1.9 |
|  | 30-64 | 16.8 | (1.9) | 19.5 | (1.5) | 21.8 | (1.5) | 20.6 | (1.6) | 21.7 | (2.3) | 19.7 | (2.2) | 0.552* | 0.821* | -2.0 |
|  | ≥65 | 3.0 | (0.9) | 3.6 | (0.7) | 4.1 | (0.6) | 6.0 | (0.9) | 7.5 | (1.5) | 4.7 | (1.1) | 0.360* | 0.455* | -2.8 |
|  | Men |  |  |  |  |  |  |  |  |  |  |  |  |  |  |  |
|  | ≥30 | 20.8 | (2.2) | 22.8 | (1.7) | 24.1 | (1.6) | 24.8 | (1.7) | 26.2 | (2.5) | 23.8 | (2.3) | 0.622* | 0.862* | -2.4 |
|  | 30-64 | 25.9 | (2.9) | 28.8 | (2.3) | 31.0 | (2.1) | 30.3 | (2.4) | 31.3 | (3.4) | 29.4 | (3.3) | 0.579 | 0.860* | -1.9 |
|  | ≥65 | 6.6 | (2.0) | 7.6 | (1.6) | 8.6 | (1.3) | 13.2 | (2.0) | 16.0 | (3.3) | 10.7 | (2.4) | 0.723* | 0.846* | -5.2 |
|  | Women |  |  |  |  |  |  |  |  |  |  |  |  |  |  |  |
|  | ≥30 | 1.8 | (0.9) | 2.7 | (0.7) | 3.2 | (0.6) | 1.9 | (0.6) | 3.1 | (1.1) | 0.9 | (0.4) | 0.057 | 0.241 | -2.2 |
|  | 30-64 | 2.9 | (1.5) | 4.4 | (1.3) | 5.7 | (1.2) | 3.5 | (1.0) | 5.6 | (2.0) | 1.8 | (0.8) | 0.089 | 0.374 | -3.8 |
|  | ≥65 | 0.3 | (0.3) | 0.6 | (0.4) | 0.7 | (0.3) | 0.4 | (0.3) | 0.8 | (0.6) | 0.0 | (0.0) | 0.009 | 0.073 | -0.8 |
| Aerobic physical activity | Total |  |  |  |  |  |  |  |  |  |  |  |  |  |  |  |
|  | ≥30 | - | - | 45.3 | (1.8) | 36.7 | (1.3) | 38.4 | (1.5) | 37.2 | (2.0) | 39.5 | (2.3) | -1.078* | -1.833*** | 2.3 |
|  | 30-64 | - | - | 50.1 | (2.4) | 42.6 | (1.8) | 41.9 | (2.1) | 39.1 | (2.7) | 44.4 | (3.2) | -1.437* | -2.476*** | 5.3 |
|  | ≥65 | - | - | 36.3 | (2.3) | 27.8 | (1.6) | 32.7 | (1.7) | 34.5 | (2.6) | 31.0 | (2.4) | -0.456 | -0.694 | -3.5 |
|  | Men |  |  |  |  |  |  |  |  |  |  |  |  |  |  |  |
|  | ≥30 | - | - | 50.4 | (2.5) | 41.4 | (1.7) | 41.0 | (2.1) | 39.0 | (2.7) | 42.7 | (3.1) | -1.896* | -3.036*** | 3.7 |
|  | 30-64 | - | - | 52.4 | (3.3) | 43.5 | (2.2) | 41.1 | (2.7) | 38.3 | (3.6) | 43.6 | (3.9) | -2.352* | -3.668*** | 5.3 |
|  | ≥65 | - | - | 45.3 | (3.2) | 36.8 | (2.4) | 40.7 | (2.8) | 40.5 | (3.7) | 40.8 | (4.2) | -0.985 | -1.854 | 0.3 |
|  | Women |  |  |  |  |  |  |  |  |  |  |  |  |  |  |  |
|  | ≥30 | - | - | 38.8 | (2.3) | 31.0 | (1.8) | 35.0 | (2.1) | 34.9 | (3.0) | 35.1 | (3.0) | 0.028 | -0.205 | 0.2 |
|  | 30-64 | - | - | 46.4 | (3.4) | 41.1 | (2.7) | 43.3 | (3.0) | 40.4 | (4.5) | 45.7 | (4.1) | 0.041 | -0.564 | 5.3 |
|  | ≥65 | - | - | 28.5 | (3.1) | 20.8 | (1.9) | 25.9 | (2.3) | 29.4 | (3.6) | 22.4 | (2.9) | -0.086 | 0.223 | -7.0 |
| Perceived Stress | Total |  |  |  |  |  |  |  |  |  |  |  |  |  |  |  |
|  | ≥30 | 22.3 | (1.5) | 24.4 | (1.2) | 23.6 | (1.0) | 25.1 | (1.3) | 24.1 | (1.8) | 26.0 | (1.9) | 0.417 | 0.339 | 1.9 |
|  | 30-64 | 23.5 | (2.0) | 26.7 | (1.6) | 26.8 | (1.4) | 30.4 | (1.8) | 28.2 | (2.4) | 32.2 | (2.6) | 0.854* | 0.658* | 4.0 |
|  | ≥65 | 19.9 | (2.1) | 20.3 | (1.8) | 18.7 | (1.3) | 17.3 | (1.5) | 18.3 | (2.2) | 16.3 | (2.0) | -0.354* | -0.242 | -2.0 |
|  | Men |  |  |  |  |  |  |  |  |  |  |  |  |  |  |  |
|  | ≥30 | 18.3 | (2.0) | 22.3 | (1.6) | 22.0 | (1.4) | 25.8 | (1.8) | 23.5 | (2.5) | 27.6 | (2.7) | 0.871* | 0.669* | 4.1 |
|  | 30-64 | 21.7 | (2.6) | 25.5 | (2.2) | 25.9 | (1.9) | 32.3 | (2.4) | 29.8 | (3.2) | 34.3 | (3.6) | 1.090* | 0.750 | 4.5 |
|  | ≥65 | 9.1 | (2.0) | 14.1 | (2.3) | 13.4 | (1.7) | 11.7 | (1.7) | 11.0 | (2.3) | 12.2 | (2.5) | 0.244 | 0.377 | 1.2 |
|  | Women |  |  |  |  |  |  |  |  |  |  |  |  |  |  |  |
|  | ≥30 | 27.0 | (2.1) | 26.9 | (1.8) | 25.5 | (1.5) | 24.4 | (1.6) | 24.8 | (2.5) | 23.9 | (2.0) | -0.141* | -0.033 | -0.9 |
|  | 30-64 | 26.3 | (3.0) | 28.5 | (2.4) | 28.3 | (2.3) | 27.1 | (2.5) | 25.7 | (3.5) | 28.4 | (3.5) | 0.419 | 0.496 | 2.8 |
|  | ≥65 | 28.0 | (3.1) | 25.1 | (2.5) | 22.8 | (2.0) | 21.7 | (2.1) | 24.0 | (3.4) | 19.5 | (2.6) | -0.713* | -0.585* | -4.5 |
| Excessive sodium intake | Total |  |  |  |  |  |  |  |  |  |  |  |  |  |  |  |
|  | ≥30 | 83.4 | (1.3) | 76.3 | (1.2) | 70.7 | (1.2) | 73.0 | (1.3) | 73.5 | (1.8) | 72.6 | (1.9) | -0.948*** | -1.198*** | -0.8 |
|  | 30-64 | 90.0 | (1.4) | 82.9 | (1.5) | 77.8 | (1.6) | 81.2 | (1.6) | 83.3 | (2.2) | 79.6 | (2.3) | -1.019*** | -1.228*** | -3.8 |
|  | ≥65 | 70.6 | (2.4) | 65.5 | (1.9) | 61.0 | (1.7) | 62.1 | (1.9) | 61.5 | (2.5) | 62.7 | (2.7) | -0.724* | -0.985* | 1.1 |
|  | Men |  |  |  |  |  |  |  |  |  |  |  |  |  |  |  |
|  | ≥30 | 90.1 | (1.4) | 85.9 | (1.3) | 82.1 | (1.3) | 84.3 | (1.6) | 87.8 | (1.8) | 81.6 | (2.3) | -0.498* | -0.371* | -6.2* |
|  | 30-64 | 93.2 | (1.6) | 88.9 | (1.6) | 86.7 | (1.6) | 89.6 | (1.8) | 93.7 | (1.9) | 86.5 | (2.7) | -0.336* | -0.204 | -7.2* |
|  | ≥65 | 81.8 | (3.1) | 79.0 | (2.3) | 73.6 | (2.2) | 75.0 | (2.6) | 78.4 | (3.3) | 71.9 | (3.8) | -0.720 | -0.550 | -6.5 |
|  | Women |  |  |  |  |  |  |  |  |  |  |  |  |  |  |  |
|  | ≥30 | 75.3 | (2.1) | 64.4 | (1.9) | 56.5 | (1.7) | 58.6 | (2.0) | 55.9 | (2.9) | 60.8 | (2.8) | -1.699*** | -2.401*** | 4.8 |
|  | 30-64 | 85.1 | (2.5) | 73.2 | (2.6) | 63.1 | (2.8) | 66.9 | (3.0) | 66.7 | (4.3) | 67.1 | (4.1) | -2.391*** | -3.090*** | 0.4 |
|  | ≥65 | 62.2 | (3.2) | 54.1 | (2.7) | 50.0 | (2.3) | 50.8 | (2.5) | 46.5 | (3.5) | 54.7 | (3.4) | -0.948* | -1.606* | 8.3 |
| Excessive energy and fat intake | Total |  |  |  |  |  |  |  |  |  |  |  |  |  |  |  |
|  | ≥30 | 2.8 | (0.6) | 3.1 | (0.6) | 3.9 | (0.6) | 5.0 | (0.8) | 4.8 | (1.0) | 5.1 | (1.1) | 0.294* | 0.264* | 0.2 |
|  | 30-64 | 3.5 | (0.9) | 4.1 | (0.8) | 5.4 | (0.9) | 7.2 | (1.2) | 7.4 | (1.7) | 7.0 | (1.7) | 0.411* | 0.373 | -0.4 |
|  | ≥65 | 1.5 | (0.5) | 1.6 | (0.5) | 1.9 | (0.6) | 2.0 | (0.6) | 1.8 | (0.8) | 2.3 | (0.9) | 0.085 | 0.070 | 0.5 |
|  | Men |  |  |  |  |  |  |  |  |  |  |  |  |  |  |  |
|  | ≥30 | 3.4 | (0.9) | 4.0 | (0.9) | 5.6 | (0.9) | 7.1 | (1.3) | 7.5 | (1.7) | 6.9 | (1.8) | 0.495* | 0.520* | -0.6 |
|  | 30-64 | 3.9 | (1.3) | 5.4 | (1.2) | 7.1 | (1.3) | 9.4 | (1.7) | 10.6 | (2.5) | 8.6 | (2.4) | 0.598* | 0.649* | -2.0 |
|  | ≥65 | 2.0 | (0.9) | 1.0 | (0.4) | 2.7 | (1.0) | 3.0 | (1.0) | 2.5 | (1.3) | 3.4 | (1.6) | 0.206 | 0.179 | 0.9 |
|  | Women |  |  |  |  |  |  |  |  |  |  |  |  |  |  |  |
|  | ≥30 | 2.2 | (0.7) | 2.0 | (0.6) | 1.9 | (0.5) | 2.2 | (0.7) | 1.6 | (0.8) | 2.7 | (1.1) | 0.007 | -0.080 | 1.1 |
|  | 30-64 | 2.9 | (1.1) | 2.0 | (0.9) | 2.6 | (0.9) | 3.3 | (1.3) | 2.3 | (1.3) | 4.2 | (2.0) | 0.058 | -0.110 | 1.9 |
|  | ≥65 | 1.2 | (0.6) | 2.1 | (0.7) | 1.2 | (0.6) | 1.2 | (0.6) | 1.1 | (0.9) | 1.3 | (0.7) | -0.022 | -0.040 | 0.2 |

Values are presented as weighted % (standard error) adjusted for age and household income level.

* p<0.05, *** p<0.001

Obesity: percentage of adults who have body mass index(BMI)≥25 kg/m^2^.

Current cigarette smoking: percentage of adults who have smoked at least 100 cigarettes during their lifetime and who are currently smokers.

High-risk alcohol drinking: percentage of adults who drink at least twice a week, with an average consumption of 7 drinks or more for men and 5 drinks or more for women.

Aerobic physical activity (PA): percentage of adults who have performed 150 minutes of moderate-intensity or 75 minutes of vigorous-intensity PA or an equivalent combination of moderate- and vigorous-intensity PA in a typical week (introduced in 2014 Korea National Health and Nutrition Examination Survey).

Perceived stress: percentage of adults who feel extremely or very stressed in their average daily life.

Excessive sodium intake: percentage of adults who consumed ≥ sodium of Intake Goal (Dietary Reference Intakes for Koreans).

Excessive energy and fat intake: percentage of adults who consumed ≥125% energy of the Estimated Energy Requirement and > fat intake of the Acceptable Macronutrient Distribution Ranges (Dietary Reference Intakes for Koreans).
