# Supplementary material for: Changes in the management of hypertension, diabetes mellitus, and hypercholesterolemia in Korean adults before and during the COVID-19 pandemic: data from the 2010-2020 Korea National Health and Nutrition Examination Survey
Source: Epidemiol Health. 2023 Feb 1;45:e2023014. doi: 10.4178/epih.e2023014 (PMC10581891; doi:10.4178/epih.e2023014)
Supplement: Supplementary Material 3. — Trends in risk factors among Korean men and women with hypercholesterolemia in the 2010-2020 Korea National Health and Nutrition Examination Survey [file epih-45-e2023014-Supplementary-3.docx]

**Supplementary Material 3. Trends in risk factors among Korean men and women with hypercholesterolemia in the 2010-2020 Korea National Health and Nutrition Examination Survey**

| **Indicators** | **Variables** | **2010-2012** | | **2013-2015** | | **2016-2018** | |  | |  | | | | **Trend** | | **Difference**  **2019 to 2020** |
| --- | --- | --- | --- | --- | --- | --- | --- | --- | --- | --- | --- | --- | --- | --- | --- | --- |
|  |  |  |  |  |  |  |  | **2019-2020** | | **2019** | | **2020** | | **2010-2020**  **(β estimate)** | **2010-2019**  **(β estimate)** |  |
| Obesity | Total |  |  |  |  |  |  |  |  |  |  |  |  |  |  |  |
|  | ≥30 | 49.6 | (1.4) | 45.6 | (1.2) | 47.5 | (1.0) | 46.1 | (1.1) | 44.3 | (1.6) | 47.7 | (1.5) | -0.129 | -0.285 | 3.3 |
|  | 30-64 | 51.3 | (1.6) | 46.9 | (1.5) | 48.1 | (1.2) | 47.3 | (1.5) | 44.8 | (2.2) | 49.6 | (2.0) | -0.185 | -0.423 | 4.8 |
|  | ≥65 | 44.1 | (2.3) | 42.3 | (1.9) | 46.1 | (1.6) | 43.4 | (1.6) | 43.3 | (2.3) | 43.5 | (2.3) | 0.020 | 0.085 | 0.2 |
|  | Men |  |  |  |  |  |  |  |  |  |  |  |  |  |  |  |
|  | ≥30 | 52.4 | (2.2) | 50.5 | (2.0) | 53.1 | (1.6) | 53.9 | (1.8) | 52.0 | (2.7) | 55.6 | (2.4) | 0.600* | 0.396 | 3.6 |
|  | 30-64 | 55.3 | (2.5) | 54.4 | (2.2) | 55.4 | (1.8) | 56.3 | (2.1) | 53.4 | (3.2) | 58.8 | (2.7) | 0.380 | 0.111 | 5.4 |
|  | ≥65 | 35.9 | (3.8) | 32.5 | (3.4) | 43.5 | (2.7) | 46.0 | (3.2) | 46.9 | (4.2) | 45.3 | (4.8) | 1.603* | 1.714* | -1.6 |
|  | Women |  |  |  |  |  |  |  |  |  |  |  |  |  |  |  |
|  | ≥30 | 47.5 | (1.6) | 42.0 | (1.5) | 42.9 | (1.2) | 39.5 | (1.2) | 38.5 | (1.7) | 40.6 | (1.7) | -0.778*** | -0.847*** | 2.1 |
|  | 30-64 | 47.6 | (1.9) | 39.8 | (2.0) | 40.6 | (1.6) | 38.0 | (1.7) | 36.6 | (2.3) | 39.3 | (2.6) | -0.871* | -1.024* | 2.7 |
|  | ≥65 | 47.2 | (2.8) | 46.3 | (2.3) | 47.3 | (1.9) | 42.1 | (1.8) | 41.6 | (2.6) | 42.6 | (2.4) | -0.616 | -0.538 | 1.0 |
| Current cigarette smoking | Total |  |  |  |  |  |  |  |  |  |  |  |  |  |  |  |
|  | ≥30 | 22.5 | (1.2) | 19.2 | (1.0) | 18.4 | (0.8) | 17.2 | (0.9) | 16.7 | (1.3) | 17.7 | (1.4) | -0.238 | -0.319 | 1.0 |
|  | 30-64 | 26.3 | (1.4) | 23.6 | (1.3) | 22.7 | (1.1) | 21.6 | (1.2) | 20.8 | (1.7) | 22.2 | (1.8) | -0.295 | -0.369 | 1.4 |
|  | ≥65 | 9.8 | (1.3) | 7.1 | (1.0) | 7.3 | (0.9) | 7.8 | (1.0) | 7.5 | (1.5) | 8.1 | (1.4) | -0.096 | -0.191 | 0.6 |
|  | Men |  |  |  |  |  |  |  |  |  |  |  |  |  |  |  |
|  | ≥30 | 44.0 | (2.1) | 39.0 | (2.0) | 35.8 | (1.6) | 33.4 | (1.7) | 33.8 | (2.5) | 33.0 | (2.4) | -0.818* | -0.864* | -0.8 |
|  | 30-64 | 47.1 | (2.4) | 43.8 | (2.4) | 40.0 | (1.8) | 38.4 | (2.0) | 38.9 | (2.9) | 38.0 | (2.8) | -0.859* | -0.869* | -0.9 |
|  | ≥65 | 25.9 | (3.6) | 16.9 | (2.7) | 18.3 | (2.3) | 17.4 | (2.6) | 17.1 | (3.9) | 17.6 | (3.6) | -0.647 | -0.833 | 0.5 |
|  | Women |  |  |  |  |  |  |  |  |  |  |  |  |  |  |  |
|  | ≥30 | 6.1 | (0.9) | 4.0 | (0.6) | 4.2 | (0.5) | 4.0 | (0.5) | 4.1 | (0.7) | 4.0 | (0.8) | -0.069 | -0.088 | 0.0 |
|  | 30-64 | 7.1 | (1.2) | 4.5 | (0.8) | 5.3 | (0.8) | 4.7 | (0.7) | 4.6 | (1.0) | 4.8 | (1.1) | -0.056 | -0.066 | 0.2 |
|  | ≥65 | 3.7 | (1.1) | 3.0 | (0.9) | 2.2 | (0.5) | 2.9 | (0.6) | 3.1 | (0.9) | 2.8 | (0.9) | -0.089 | -0.125 | -0.4 |
| High-risk alcohol drinking | Total |  |  |  |  |  |  |  |  |  |  |  |  |  |  |  |
|  | ≥30 | 12.9 | (1.0) | 10.9 | (0.8) | 12.6 | (0.7) | 12.5 | (0.8) | 11.3 | (1.1) | 13.7 | (1.1) | 0.252 | 0.131 | 2.4 |
|  | 30-64 | 16.1 | (1.2) | 14.0 | (1.1) | 16.3 | (0.9) | 15.8 | (1.1) | 13.3 | (1.5) | 18.1 | (1.5) | 0.207 | 0.028 | 4.8* |
|  | ≥65 | 2.5 | (0.6) | 2.4 | (0.5) | 3.1 | (0.5) | 5.5 | (0.8) | 6.8 | (1.2) | 4.3 | (1.0) | 0.365*** | 0.418*** | -2.5 |
|  | Men |  |  |  |  |  |  |  |  |  |  |  |  |  |  |  |
|  | ≥30 | 25.6 | (1.9) | 21.0 | (1.7) | 23.9 | (1.4) | 24.2 | (1.6) | 21.7 | (2.3) | 26.2 | (2.1) | 0.280 | 0.027 | 4.5 |
|  | 30-64 | 28.6 | (2.2) | 24.1 | (2.0) | 27.6 | (1.7) | 27.4 | (2.0) | 23.1 | (2.9) | 31.0 | (2.6) | 0.176 | -0.146 | 7.9* |
|  | ≥65 | 8.0 | (1.9) | 6.9 | (1.6) | 8.8 | (1.5) | 14.0 | (2.1) | 17.3 | (3.2) | 11.5 | (2.6) | 0.762* | 0.895* | -5.8 |
|  | Women |  |  |  |  |  |  |  |  |  |  |  |  |  |  |  |
|  | ≥30 | 3.3 | (0.8) | 3.2 | (0.6) | 3.4 | (0.5) | 3.0 | (0.5) | 3.6 | (0.8) | 2.5 | (0.6) | 0.042 | 0.093 | -1.1 |
|  | 30-64 | 4.5 | (1.0) | 4.6 | (0.9) | 5.0 | (0.7) | 4.2 | (0.7) | 4.5 | (1.0) | 3.9 | (0.9) | 0.026 | 0.070 | -0.6 |
|  | ≥65 | 0.4 | (0.3) | 0.5 | (0.3) | 0.5 | (0.2) | 1.1 | (0.4) | 2.0 | (0.8) | 0.3 | (0.2) | 0.073 | 0.135 | -1.7 |
| Aerobic physical activity | Total |  |  |  |  |  |  |  |  |  |  |  |  |  |  |  |
|  | ≥30 | - | - | 48.1 | (1.6) | 40.4 | (1.0) | 41.2 | (1.1) | 39.1 | (1.5) | 43.2 | (1.7) | -1.126* | -2.207*** | 4.1 |
|  | 30-64 | - | - | 51.3 | (1.9) | 43.4 | (1.3) | 44.4 | (1.4) | 40.2 | (1.8) | 48.5 | (2.1) | -1.067* | -2.631*** | 8.3* |
|  | ≥65 | - | - | 39.5 | (2.4) | 32.6 | (1.5) | 33.8 | (1.7) | 36.9 | (2.4) | 30.7 | (2.4) | -1.198* | -1.045 | -6.1 |
|  | Men |  |  |  |  |  |  |  |  |  |  |  |  |  |  |  |
|  | ≥30 | - | - | 52.2 | (2.6) | 44.1 | (1.5) | 44.0 | (1.8) | 42.2 | (2.5) | 45.6 | (2.6) | -1.300* | -2.326* | 3.4 |
|  | 30-64 | - | - | 52.9 | (2.9) | 45.7 | (1.8) | 45.3 | (2.1) | 42.1 | (2.9) | 48.0 | (2.9) | -1.174 | -2.385* | 5.9 |
|  | ≥65 | - | - | 48.8 | (4.4) | 37.5 | (2.7) | 39.6 | (3.2) | 42.5 | (5.0) | 37.0 | (4.4) | -1.761 | -2.154 | -5.5 |
|  | Women |  |  |  |  |  |  |  |  |  |  |  |  |  |  |  |
|  | ≥30 | - | - | 44.9 | (1.9) | 37.5 | (1.3) | 38.9 | (1.4) | 36.9 | (1.9) | 41.1 | (2.1) | -1.018* | -2.111*** | 4.2 |
|  | 30-64 | - | - | 49.7 | (2.4) | 41.2 | (1.7) | 43.6 | (1.9) | 38.4 | (2.4) | 49.0 | (2.8) | -0.972 | -2.857*** | 10.6* |
|  | ≥65 | - | - | 35.9 | (2.8) | 30.4 | (1.7) | 30.9 | (1.9) | 34.3 | (2.7) | 27.3 | (2.7) | -1.057 | -0.679 | -7.0 |
| Perceived Stress | Total |  |  |  |  |  |  |  |  |  |  |  |  |  |  |  |
|  | ≥30 | 25.9 | (1.1) | 24.1 | (1.1) | 25.3 | (0.9) | 27.2 | (1.0) | 26.8 | (1.3) | 27.5 | (1.4) | 0.370* | 0.238 | 0.6 |
|  | 30-64 | 26.7 | (1.4) | 25.9 | (1.4) | 26.8 | (1.1) | 31.0 | (1.3) | 29.6 | (1.8) | 32.2 | (1.9) | 0.602* | 0.354 | 2.5 |
|  | ≥65 | 23.2 | (1.9) | 18.9 | (1.5) | 21.5 | (1.3) | 19.1 | (1.3) | 20.7 | (2.0) | 17.6 | (1.7) | -0.342 | -0.155 | -3.2 |
|  | Men |  |  |  |  |  |  |  |  |  |  |  |  |  |  |  |
|  | ≥30 | 20.7 | (1.7) | 23.6 | (1.8) | 24.3 | (1.4) | 29.9 | (1.6) | 30.1 | (2.3) | 29.8 | (2.2) | 1.296*** | 1.156*** | -0.3 |
|  | 30-64 | 22.0 | (2.0) | 26.4 | (2.1) | 26.8 | (1.7) | 35.1 | (2.0) | 34.6 | (2.8) | 35.5 | (2.7) | 1.518*** | 1.307*** | 0.9 |
|  | ≥65 | 13.0 | (2.8) | 10.2 | (2.0) | 13.7 | (1.8) | 13.3 | (2.1) | 14.7 | (3.2) | 12.2 | (2.6) | 0.236 | 0.410 | -2.6 |
|  | Women |  |  |  |  |  |  |  |  |  |  |  |  |  |  |  |
|  | ≥30 | 29.9 | (1.5) | 24.5 | (1.3) | 26.1 | (1.1) | 24.9 | (1.1) | 24.5 | (1.6) | 25.4 | (1.7) | -0.322 | -0.438 | 0.9 |
|  | 30-64 | 31.0 | (1.9) | 25.5 | (1.6) | 26.7 | (1.4) | 26.8 | (1.5) | 25.1 | (2.0) | 28.5 | (2.4) | -0.289 | -0.546 | 3.4 |
|  | ≥65 | 27.1 | (2.3) | 22.5 | (1.9) | 25.1 | (1.6) | 22.0 | (1.7) | 23.4 | (2.6) | 20.6 | (2.3) | -0.462 | -0.302 | -2.9 |
| Excessive sodium intake | Total |  |  |  |  |  |  |  |  |  |  |  |  |  |  |  |
|  | ≥30 | 85.5 | (0.9) | 74.9 | (1.1) | 70.6 | (1.0) | 71.7 | (1.1) | 71.2 | (1.5) | 72.2 | (1.6) | -1.260*** | -1.569*** | 1.0 |
|  | 30-64 | 89.5 | (1.0) | 78.9 | (1.4) | 75.8 | (1.1) | 75.8 | (1.3) | 75.3 | (1.8) | 76.4 | (1.9) | -1.396*** | -1.672*** | 1.1 |
|  | ≥65 | 72.0 | (2.0) | 64.9 | (1.8) | 58.6 | (1.6) | 63.2 | (1.9) | 62.8 | (2.3) | 63.5 | (2.7) | -0.814* | -1.219*** | 0.7 |
|  | Men |  |  |  |  |  |  |  |  |  |  |  |  |  |  |  |
|  | ≥30 | 93.6 | (1.0) | 87.0 | (1.4) | 86.8 | (1.0) | 84.0 | (1.4) | 84.4 | (1.9) | 83.7 | (2.0) | -0.798*** | -0.800*** | -0.7 |
|  | 30-64 | 95.6 | (1.0) | 88.7 | (1.6) | 89.9 | (1.1) | 85.7 | (1.6) | 86.0 | (2.3) | 85.6 | (2.2) | -0.882*** | -0.839*** | -0.4 |
|  | ≥65 | 82.0 | (3.3) | 79.9 | (3.0) | 76.5 | (2.5) | 78.8 | (2.9) | 79.8 | (3.8) | 78.0 | (4.1) | -0.257 | -0.388 | -1.8 |
|  | Women |  |  |  |  |  |  |  |  |  |  |  |  |  |  |  |
|  | ≥30 | 79.1 | (1.4) | 65.5 | (1.5) | 57.5 | (1.3) | 61.5 | (1.5) | 61.2 | (2.0) | 61.8 | (2.2) | -1.831*** | -2.293*** | 0.6 |
|  | 30-64 | 83.7 | (1.6) | 69.3 | (2.0) | 62.1 | (1.7) | 65.8 | (1.9) | 65.4 | (2.4) | 66.1 | (2.9) | -2.044*** | -2.506*** | 0.6 |
|  | ≥65 | 68.0 | (2.3) | 58.5 | (2.2) | 49.7 | (1.9) | 54.8 | (2.2) | 54.4 | (2.9) | 55.3 | (3.2) | -1.329*** | -1.803*** | 0.8 |
| Excessive energy and fat intake | Total |  |  |  |  |  |  |  |  |  |  |  |  |  |  |  |
|  | ≥30 | 3.2 | (0.6) | 3.7 | (0.5) | 4.3 | (0.5) | 4.8 | (0.6) | 4.6 | (0.7) | 5.1 | (0.9) | 0.244* | 0.223* | 0.5 |
|  | 30-64 | 3.7 | (0.7) | 4.4 | (0.7) | 5.3 | (0.7) | 6.1 | (0.8) | 6.1 | (1.0) | 6.1 | (1.1) | 0.283* | 0.281* | 0.0 |
|  | ≥65 | 1.5 | (0.5) | 1.6 | (0.6) | 1.9 | (0.5) | 2.3 | (0.7) | 1.5 | (0.6) | 3.0 | (1.2) | 0.128 | 0.053 | 1.5 |
|  | Men |  |  |  |  |  |  |  |  |  |  |  |  |  |  |  |
|  | ≥30 | 5.5 | (1.2) | 5.2 | (1.0) | 6.9 | (1.0) | 8.4 | (1.2) | 7.8 | (1.5) | 8.8 | (1.8) | 0.419* | 0.353 | 1.0 |
|  | 30-64 | 5.7 | (1.3) | 6.1 | (1.2) | 8.1 | (1.2) | 9.6 | (1.4) | 9.6 | (2.0) | 9.6 | (2.1) | 0.473* | 0.459* | 0.0 |
|  | ≥65 | 4.4 | (1.6) | 1.6 | (0.8) | 3.1 | (1.1) | 4.6 | (1.7) | 2.6 | (1.5) | 6.4 | (2.9) | 0.194 | -0.098 | 3.9 |
|  | Women |  |  |  |  |  |  |  |  |  |  |  |  |  |  |  |
|  | ≥30 | 1.3 | (0.4) | 2.4 | (0.5) | 2.2 | (0.3) | 1.9 | (0.4) | 2.1 | (0.6) | 1.7 | (0.6) | 0.071 | 0.107 | -0.4 |
|  | 30-64 | 1.7 | (0.5) | 2.8 | (0.7) | 2.6 | (0.5) | 2.5 | (0.6) | 2.8 | (1.0) | 2.2 | (0.8) | 0.064 | 0.103 | -0.7 |
|  | ≥65 | 0.3 | (0.3) | 1.6 | (0.7) | 1.4 | (0.4) | 1.0 | (0.5) | 1.0 | (0.6) | 1.1 | (0.7) | 0.073 | 0.101 | 0.0 |

Values are presented as weighted % (standard error) adjusted for age and household income level.

* p<0.05, *** p<0.001

Obesity: percentage of adults who have body mass index(BMI)≥25 kg/m^2^.

Current cigarette smoking: percentage of adults who have smoked at least 100 cigarettes during their lifetime and who are currently smokers.

High-risk alcohol drinking: percentage of adults who drink at least twice a week, with an average consumption of 7 drinks or more for men and 5 drinks or more for women.

Aerobic physical activity (PA): percentage of adults who have performed 150 minutes of moderate-intensity or 75 minutes of vigorous-intensity PA or an equivalent combination of moderate- and vigorous-intensity PA in a typical week (introduced in 2014 Korea National Health and Nutrition Examination Survey).

Perceived stress: percentage of adults who feel extremely or very stressed in their average daily life.

Excessive sodium intake: percentage of adults who consumed ≥ sodium of Intake Goal (Dietary Reference Intakes for Koreans).

Excessive energy and fat intake: percentage of adults who consumed ≥125% energy of the Estimated Energy Requirement and > fat intake of the Acceptable Macronutrient Distribution Ranges (Dietary Reference Intakes for Koreans).
